# Blood pressure variability, nocturnal heart rate variability and endothelial function predict recurrent cerebro-cardiovascular events following ischemic stroke

**DOI:** 10.3389/fcvm.2023.1288109

**Published:** 2023-11-16

**Authors:** Irina Filchenko, Nicolas Mürner, Martijn P. J. Dekkers, Sebastien Baillieul, Simone B. Duss, Anne-Kathrin Brill, Thomas Horvath, Mirjam R. Heldner, Emrush Rexhaj, Corrado Bernasconi, Claudio L. A. Bassetti, Markus H. Schmidt

**Affiliations:** ^1^ Sleep-Wake-Epilepsy Center, Department of Neurology, Inselspital, Bern University Hospital, University of Bern, Bern, Switzerland; ^2^ Graduate School for Health Sciences, University of Bern, Bern, Switzerland; ^3^ Univ. Grenoble Alpes, Inserm, U1300, CHU Grenoble Alpes, Service Universitaire de Pneumologie Physiologie, Grenoble, France; ^4^ Department of Pulmonary Medicine and Allergology, Inselspital, Bern University Hospital, University of Bern, Bern, Switzerland; ^5^ Stroke Unit, Department of Neurology, Inselspital, Bern University Hospital, University of Bern, Bern, Switzerland; ^6^ Department of Cardiology, Inselspital, Bern University Hospital, University of Bern, Bern, Switzerland; ^7^ Ohio Sleep Medicine Institute, Dublin, OH, United States

**Keywords:** blood pressure variability, heart rate variability, arterial stiffness, endothelial dysfunction, cerebro-cardiovascular risk, ischemic stroke, cardiovascular outcome, EndoPAT

## Abstract

**Introduction:**

Cardiovascular parameters characterizing blood pressure (BP), heart rate (HR), endothelial function and arterial stiffness predict cerebro-cardiovascular events (CCVE) in the general population. Considering the paucity of data in stroke patients, we assessed these parameters as potential predictors of recurrent CCVE at acute stroke stroke.

**Patients and methods:**

This is a secondary outcome analysis of a prospective observational longitudinal Sleep Deficiency & Stroke Outcome Study (ClinicalTrials.gov Identifier: NCT02559739). The study consecutively recruited acute ischemic stroke patients. Cardiovascular parameters (blood pressure variability [BPV], heart rate variability [HRV], endothelial function, and arterial stiffness) were assessed within the first week post-stroke. Future CCVE were recorded over a 3-year follow-up. Multivariate Cox regression analysis was used to investigate the prognostic value of 48 cardiovascular parameters regarding CCVE risk.

**Results:**

Out of 447 recruited patients, 359 were included in this analysis. 20% of patients developed a future CCVE. A high variability of systolic BP (*n* = 333) and nocturnal HR (non-linear parameters; *n* = 187) at acute stroke predicted CCVE risk after adjustment for demographic parameters, cardiovascular risk factors and mean BP or HR, respectively. Endothelial dysfunction (*n* = 105) at acute stroke predicted CCVE risk after adjustment for age and sex, but not after adjustment for cardiovascular risk factors. Diurnal HR and arterial stiffness at acute stroke were not associated with CCVE risk.

**Conclusion:**

High blood pressure variability, high nocturnal HRV and endothelial function contribute to the risk for future CCVE after stroke.

## Graphical Abstract

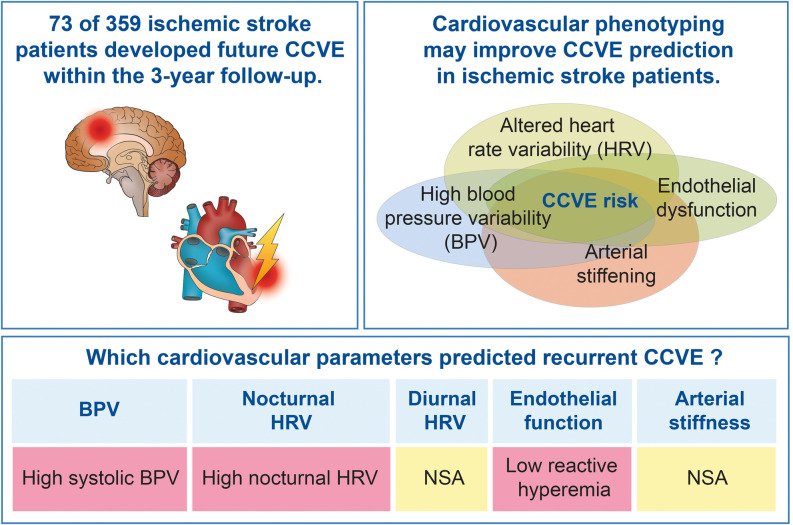

## Introduction

The incidence of cerebro-cardiovascular events (CCVE) has decreased by nearly half in recent decades, largely due to modifications in cerebro-cardiovascular risk factors (1, 2). However, the risk for CCVE remains high in certain populations, such as ischemic stroke patients.

Ischemic stroke affects 94 per 100 000 people per year worldwide (3), and following a transient ischemic attack (TIA) or a minor ischemic stroke, 6.2% of patients are affected by recurrent CCVE within one year (4). This risk escalates to an estimated cumulative rate of 12.9% over five years (5).

Current approaches for the assessment of vascular risk are based on the clinical and laboratory characteristics (e.g., lipid profile) (6). Several studies confirmed the predictive value of high blood pressure variability (BPV) regarding future CCVE in ischemic stroke patients (7, 8), while little is known about the prognostic value of heart rate variability (HRV), endothelial function and arterial stiffness in this population (9). Moreover, the properties of these parameters in stroke patients, such as the associations with demographic and vascular risk factors, are not fully understood (10, 11).

We hypothesized that altered BPV, HRV, arterial stiffness and endothelial function represent a disequilibrium in cardiovascular homeostasis resulting in the increased risk for future CCVE after stroke. In this prospective study, our primary objective was to explore the ability of cardiovascular parameters at acute stroke to predict recurrent CCVE after stroke. The secondary objective was to characterize cardiovascular parameters in a population with acute stroke.

## Methods

### Study population

This is a secondary outcome analysis of the prospective longitudinal observational cohort study “Sleep Deficiency and Stroke Outcome” (SD&SO; clinicaltrials.gov: NCT02559739). The study was conducted at the Bern University Hospital and at the Ospedale Regionale di Lugano. However, the cardiovascular assessments included in this analysis were only performed in Bern.

The study assessed patients consecutively admitted to the Department of Neurology at Bern University Hospital (recruitment period: 7/2015–1/2018) with a diagnosis of acute ischemic stroke or transient ischemic attack (TIA, within 7 days from symptom onset). Eligible patients were 18–85 years of age and able to give informed consent and to follow the study procedures. The exclusion criteria were the following: primary hemorrhagic stroke, clinically unstable or life-threatening conditions (coma/stupor, severe heart failure, oxygen-dependent pulmonary disease or severe pulmonary complications, severe renal or liver insufficiency), pregnancy, drug or alcohol abuse.

This study followed the STROBE reporting guideline for cohort studies. The protocol was approved by the Institutional Review Boards and the cantonal ethics committees. The study was conducted in compliance with the Declaration of Helsinki and Good Clinical Practice. All study participants provided written informed consent prior to participation.

### Study procedures

At hospital admission, demographics, anthropometric characteristics [body mass index (BMI)] and pre-stroke medical history were evaluated. Pre-stroke medical history included arterial hypertension (blood pressure ≥140/90 mmHg measured ≥3 times before stroke or patients under treatment for hypertension), diabetes (fasting glucose level ≥140 mg/dl or patients treated for diabetes), dyslipidemia (cholesterol level ≥250 mg/dl or previously under treatment), coronary artery disease, heart failure, smoking status, peripheral artery disease (PAD), atrial fibrillation and the symptoms of depression (according to Beck Depression Inventory, BDI).

The following stroke characteristics were assessed: stroke severity according to the National Institute of Health Stroke Scale (NIHSS; at admission and at discharge), stroke etiology according to TOAST stroke classification (12), stroke topography relative to tentorium (based on magnetic resonance imaging within clinical routine), recanalization therapy, wake-up stroke and post-stroke functional disability according to the modified Rankin Scale (mRS; at 3 months post-stroke).

The presence of sleep-disordered breathing was assessed using an overnight respiratory polygraphy (Nox T3®, Nox Medical, Reykjavík, Iceland) or a nocturnal Apnea link™ device (ResMed Bella Vista, Australia) within 7 days after stroke as part of the routine post-stroke work-up. Respiratory events were scored according to the American Academy of Sleep Medicine (AASM) criteria v2.4 (13).

### Assessment of cardiovascular parameters

The following cardiovascular parameters were assessed within the first week from stroke onset: blood pressure, nocturnal and diurnal heart rate, endothelial function, and arterial stiffness. The physiological characteristics of the cardiovascular parameters according to the current literature are summarized in Supplementary Table S1. The assessment of cardiovascular parameters is detailed in Supplementary material S1.

#### Blood pressure

BP and BPV were assessed based on in-hospital and outpatient measurements. BPV characteristics estimated mean, the deviation from a central point (standard deviation [SD], coefficient of variation [CV], mean absolute deviation [MAD]), variance, successive deviation [successive variation, average real variability (ARV)] and range (14, 15).

#### Nocturnal heart rate

Mean nocturnal HR and HRV were assessed based on the available pulse rate data from the nocturnal respiratory polygraphy. Nocturnal HRV was calculated using HRVTool, an Open-Source Matlab Toolbox for Analyzing Heart Rate Variability based on pulse-wave form (16). HRV-parameters were represented in 3 main domains: the time domain was represented by HRV based on relative RR intervals (rrHRV), standard deviation of normal-to-normal beats (SDNN), standard deviation of successive differences (SDSD), triangular index (TI), root mean square of successive differences of successive RR intervals (RMSSD), and the percentage of adjacent NN intervals that differ from each other >50 ms (pNN50). The frequency domain was represented by normalized low frequency (nuLf) power, normalized high frequency (nuHF) power, nuLF/nuHf ratio, low frequency (LF) power, high frequency (HF) power and very low frequency (VLF) power. Lastly, non-linear measurements were represented by SD1 and SD2 from Poincaré plots, SD1/SD2 ratio, detrended fluctuation analysis *α*1 (DFA1), detrended fluctuation analysis *α*2 (DFA2) and approximate entropy (ApEn).

#### EndoPAT: diurnal heart rate, endothelial function and arterial stiffness

Approximately 20% of the patients underwent additional assessments of diurnal HRV, endothelial function and arterial stiffness with digital plethysmography (EndoPAT2000-device; Itamar Medical Ltd., Caesarea, Israel).

Endothelial function, arterial stiffness and diurnal heart rate (mean and HRV) were represented as follows: (1) mean HR; (2) time domain of HRV—SDNN, RMSSD, pNN50 and TI; (3) frequency domain of HRV—LF power (LF), HF power (HF) and LF/HF; (4) endothelial function—reactive hyperemia index (RHI) and Framingham reactive hyperemia index (FRHI); (5) arterial stiffness—augmentation index and augmentation index corrected for a heart rate of 75 bpm (AI75).

### Cerebro-cardiovascular events (CCVE)

The main outcome of the study was a composite of fatal and non-fatal future CCVE that included ischemic or hemorrhagic stroke, transient ischemic attack, myocardial infarction, unplanned hospitalization for unstable angina or heart failure and urgent revascularization. CCVE were assessed by a research fellow or a study nurse in a structured telephone interview over a 3-year (1095-day) period after study inclusion (Supplementary material S2).

### Statistical analysis

**Figure 1:**
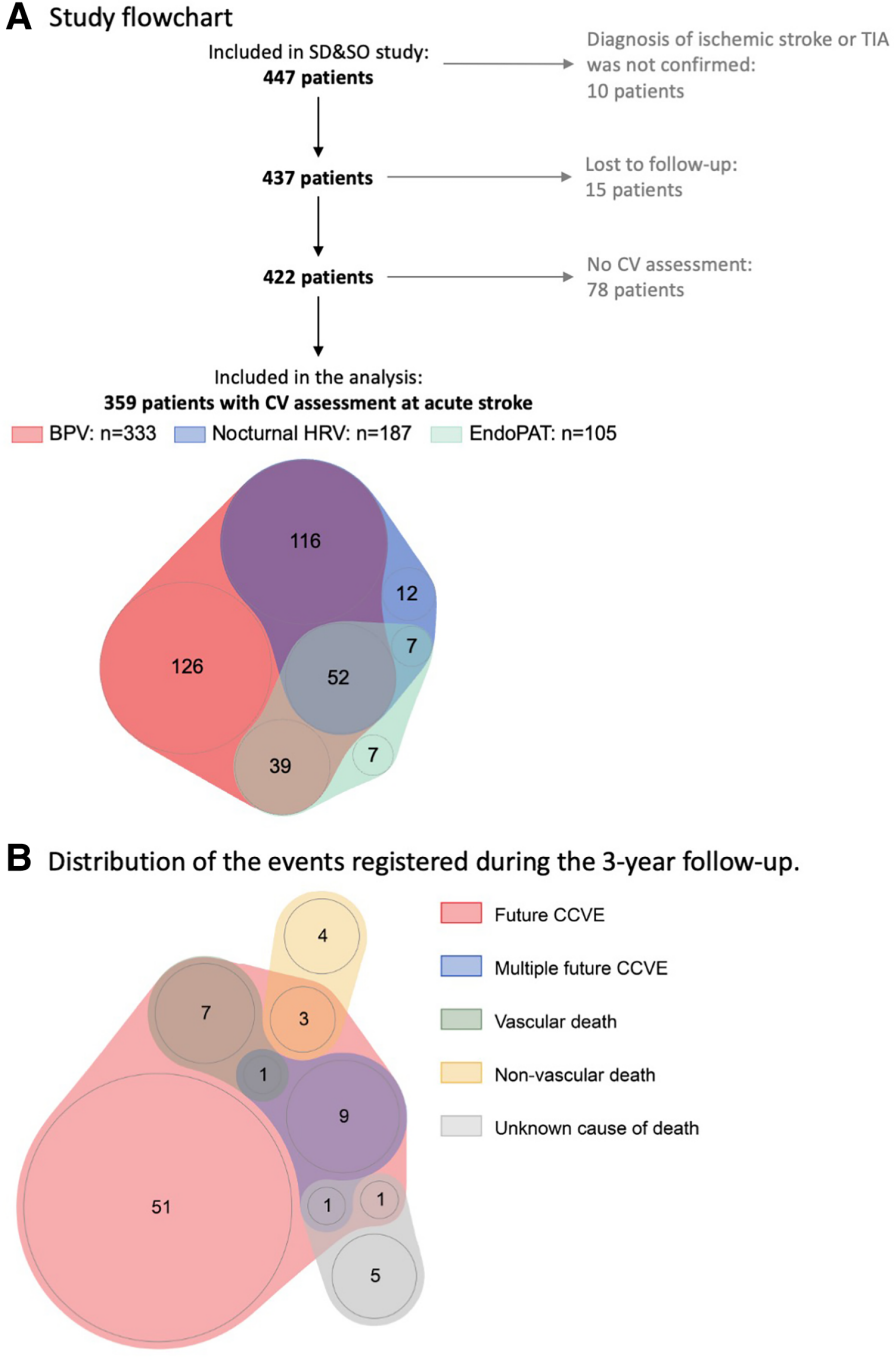
(**A**) Sleep Deficiency and Stroke Outcome study flowchart. (**B**) Distribution of the events registered during 3-year follow-up. 20% (73) patients developed 87 recurrent CCVE. EndoPAT assessments included diurnal HRV, endothelial function and arterial stiffness.

The statistical analysis was exploratory and performed using R version 4.0.1 (cran.r-project.org/). Continuous variables were presented as median and interquartile range. Categorical variables were presented as count (% of total). Only the patients with available measurements of cardiovascular characteristics and available information about CCVE risk were included in the analysis (Figure 1A). Missing values were not imputed.

We performed a descriptive analysis, and, following inspection of the distribution of continuous variables, we log-transformed non-normally distributed variables (rrHRV, SDNN, SDSD, RMSSD, pNN50). We then used Spearman's rank correlation to investigate the associations between continuous variables: cardiovascular parameters, demographics, stroke characteristics and cardiovascular risk factors. The significant associations according to correlation analysis were further investigated using multiple linear regression adjusted for age and sex. Finally, we investigated the associations between cardiovascular parameters and future CCVE within 3 years post-stroke using univariate and multiple Cox regression models. We consecutively adjusted multiple Cox regression models as follows: (1) age + sex; (2) age + sex + cardiovascular risk factors. To delineate the potential impact of mean on variability (17), multiple Cox regression models that included systolic BPV, diastolic BPV, nocturnal HRV or diurnal HRV as independent variables were additionally adjusted for mean SBP, mean DBP, mean nocturnal HR or mean diurnal HR, respectively.

Cardiovascular risk factors were selected based on World Health Organization cardiovascular disease risk charts and Framingham Risk Score and included the presence of hypertension, diabetes and dyslipidemia as well as current smoking. These cardiovascular risk factors were coded as binary variables (treated or untreated vs. absent for hypertension, diabetes and dyslipidemia; presence or absence of current smoking). Since cardiovascular parameters are associated with atrial fibrillation (AF) (18–21), we additionally adjusted models for atrial fibrillation. We compared models using the Akaike Information Criterion (AIC). All statistical tests were two-sided and conducted at the 5% significance level.

## Results

### Study population

Of the 447 patients enrolled in the SD&SO study, 359 patients were included in the analysis (Figure 1A). Patients were 67.1 [57.6, 74.7] years old and 35.9% were female. The full study population, which included the patients with rather minor stroke, is shown in Table 1 and in Supplementary Tables S2, S3.

**Table 1:** Background characteristics of study participants (*n* = 359).

Parameter	*N*	Value
Age, years	359	67.1 [57.6, 74.7]
Female sex	359	129 (35.9%)
TOAST	359	
Cardioembolism		116 (32.3%)
Large artery atherosclerosis		89 (24.8%)
Other		17 (4.7%)
Small vessel occlusion		25 (7.0%)
Unknown		112 (31.2%)
Stroke topography	359	
Supratentorial		241 (67.1%)
Infratentorial		61 (17.0%)
Both supra- and infratentorial		9 (2.5%)
Transient ischemic attack		48 (13.4%)
Wake-up stroke	359	78 (21.7%)
NIHSS at admission, score	359	2.0 [1.0, 4.0]
NIHSS at discharge, score	359	1.0 [0.0, 2.0]
mRS at 3 months post-stroke, score	282	1.0 [0.0, 1.0]
Intravascular stroke treatment	358	99 (27.7%)
Hypertension	353	
No		132 (37.4%)
Yes, treated		183 (51.8%)
Yes, untreated		38 (10.8%)
Diabetes	359	
No		300 (83.6%)
Yes, treated		52 (14.5%)
Yes, untreated		7 (1.9%)
Dyslipidemia	343	
No		141 (41.1%)
Yes, treated		101 (29.4%)
Yes, untreated		101 (29.4%)
Body mass index, kg/m^2^	358	26.3 [23.8, 29.3]
Obesity^a^	358	72 (20.1%)
Coronary artery disease	359	47 (13.1%)
Heart failure	358	10 (2.8%)
Atrial fibrillation	353	40 (11.3%)
Peripheral artery disease	358	16 (4.5%)
Current smoking	356	92 (25.8%)
Beck Depression Inventory Score	309	4.0 [2.0, 9.0]
Depression	309	75 (24.3%)
Apnea-hypopnea index	329	7.8 [3.5, 20.6]
Sleep-disordered breathing^b^	329	84 (25.5%)
Previous CCVE	359	97 (27.0%)
Future CCVE	359	73 (20.3%)

Continuous data is presented as median [interquartile range]. Categorical data is presented as count (% of total).

^a^ Defined as body mass index ≥30 kg/m^2^.

^b^ Defined as apnea-hypopnea index ≥20/h.

### Associations between cardiovascular parameters at acute stroke

The results of correlation analysis are summarized in Supplementary Figure S1. In brief, high BPV (predominantly, diastolic BPV) was associated with high HRV, endothelial dysfunction and arterial stiffening. Furthermore, high nocturnal HRV was associated with high diurnal HRV, and endothelial dysfunction was associated with arterial stiffening. Only few associations between HRV, endothelial dysfunction and arterial stiffening were significant.

### Association of cardiovascular parameters at acute stroke with demographic characteristics, stroke characteristics and comorbidities

**Figure 2:**
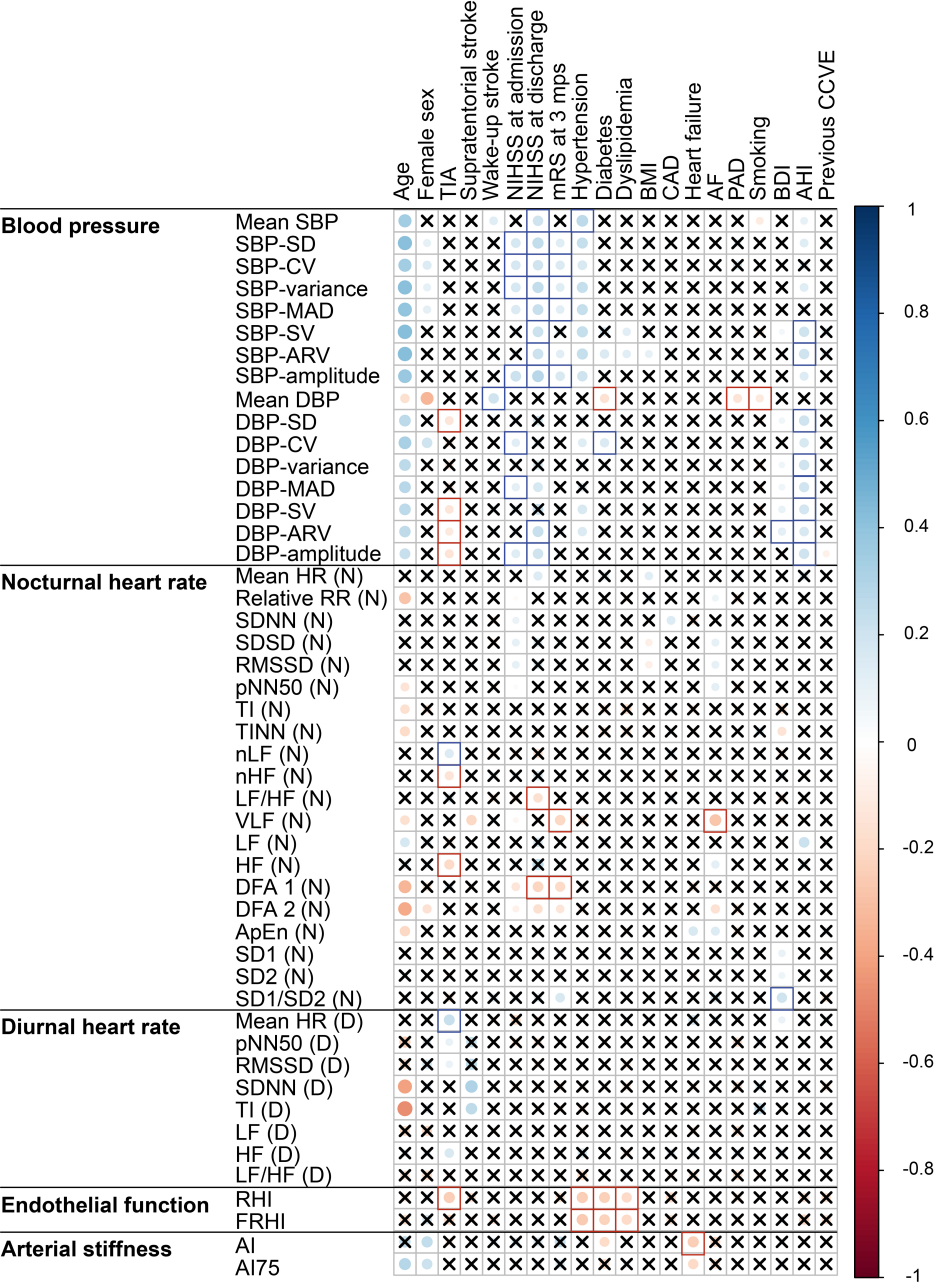
Correlation plot of the associations of cardiovascular parameters recorded at acute stroke with demographics, stroke characteristics and cardiovascular risk factors. Significant (*p* < 0.05) associations according to Spearman correlation test are shown as filled circles. Crosses mark the insignificant (*p* ≥ 0.05) associations. Blank squares mark the associations that remained significant in multiple regression models after adjustment for age and sex. The abbreviations for cardiovascular parameters are specified in the abbreviation list. AF, atrial fibrillation; bpm, beats per minute; BDI, Beck Depression Inventory; BMI, body mass index; CAD, coronary artery disease; TIA, transient ischemic attack.

At acute stroke, BP parameters were positively associated with age. Female sex was associated negatively with mean DBP and positively with coefficient of variation of DBP (DBP-CV). Both nocturnal and diurnal HRV at acute stroke were negatively associated with age, and arterial stiffness was positively associated with female sex. There were no other associations between demographic and cardiovascular parameters at acute stroke (Figure 2 and Supplementary Table S4).

High BP, BPV and nocturnal HRV (DFA1, LF/HF, VLF) at acute stroke were associated with severe neurological and functional disability at acute stroke and at 3 months post-stroke. Low diastolic BPV, HRV reflecting parasympathetic dominance (HF, nHF, LF) and good endothelial functioning were associated with TIA instead of stroke.

At acute stroke, mean systolic BP and BPV, nocturnal HRV as derived from frequency and non-linear domains, high diurnal HR, endothelial dysfunction and low arterial stiffness were associated with vascular risk factors (e.g., hypertension, diabetes and sleep-disordered breathing).

### Association of cardiovascular characteristics at acute stroke with future cerebro-cardiovascular events

**Figure 3:**
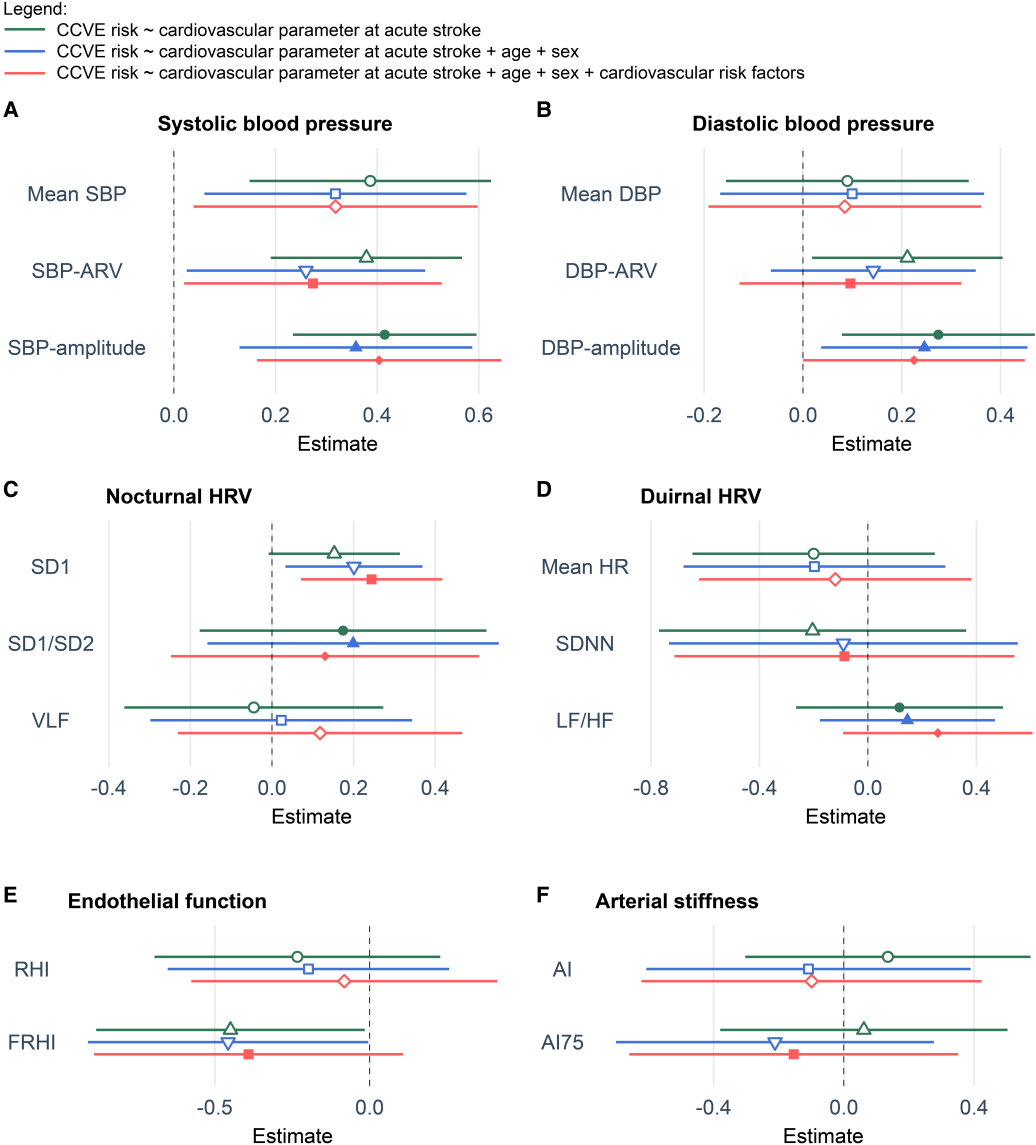
Coefficient plots of Cox regression models showing the association of cardiovascular parameters at acute stroke with the risk for future CCVE. (**A**) Systolic blood pressure. (**B**) Diastolic blood pressure. (**C**) Nocturnal heart rate. (**D**) Diurnal heart rate. (**E**) Endothelial function. (**F**) Arterial stiffness. All multivariate models including blood pressure variability or heart rate variability were respectively adjusted for mean SBP, mean DBP, mean nocturnal HR or mean diurnal HR. All values are presented as z-scores. Cardiovascular risk factors included hypertension, diabetes, dyslipidemia, current smoking and atrial fibrillation. The abbreviations for cardiovascular parameters are specified in the abbreviation list.

**Figure 4:**
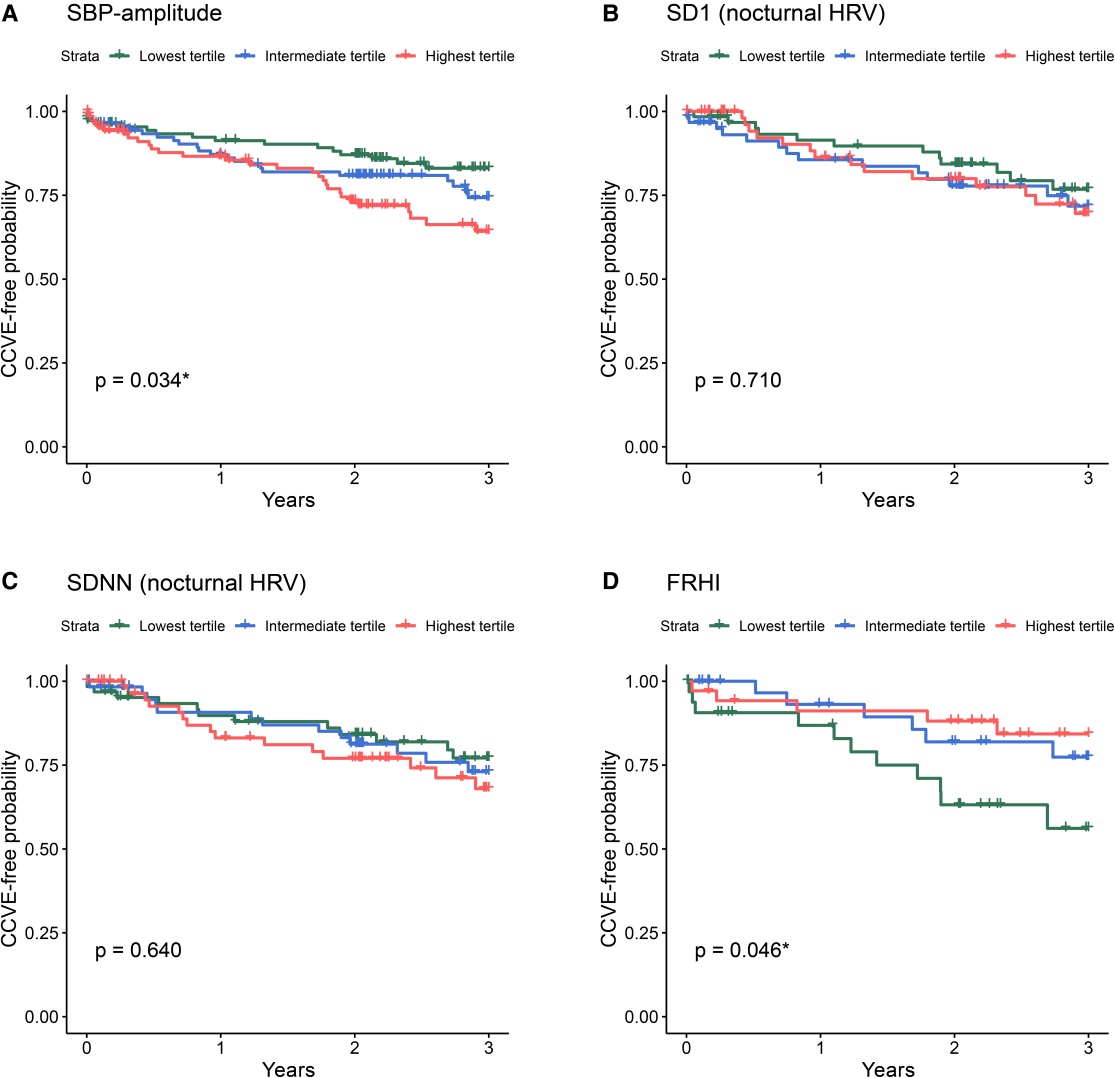
Kaplan-Meier survival curves for the risk for future CCVE according to the tertiles of selected cardiovascular parameters at acute stroke: SBP-amplitude (**A**), SD1 of nocturnal HRV (**B**), SDNN of nocturnal HRV (**C**), and FRHI (**D**). The abbreviations for cardiovascular parameters are specified in the abbreviation list.

Seventy-three of 359 stroke patients (20%) developed 87 CCVE within the 3-year follow-up period (Figure 1B). Sixty-two patients (17%) developed a single CCVE, whereas 11 (3%) patients developed multiple CCVE. During the observation period, 14 of 359 stroke patients (4%) died from non-vascular causes. Regression models are presented in detail in Figures 3, 4 and Supplementary Table S5.

Univariate Cox regression analysis (Figure 3, green lines) showed the significant association of CCVE risk with several BP parameters. There was a significant association of CCVE risk with high SDNN and a trend for a potential association of CCVE risk with high SD1 and SD2. Endothelial dysfunction (FRHI) was significantly associated with CCVE risk. This univariate analysis is partially illustrated in Kaplan-Meier survival curves for the risk for future CCVE according to the tertiles of selected cardiovascular parameters (Figure 4).

In a second step, we used multiple Cox regression models adjusted for age, sex and mean value of cardiovascular parameter (whenever applicable; Figure 3, blue lines). In these models, the associations of most BP parameters and endothelial dysfunction (FRHI) with CCVE risk remained significant. Moreover, the association of high SD1 and SD2 with CCVE risk became significant.

In a third step, we further adjusted the multiple Cox regression models by adding vascular risk factors (Figure 3, red lines). In these models, the association of SBP parameters and non-linear HRV parameters (SD1 and SD2) remained significant, while the associations of DBP parameters and FRHI with CCVE risk lost their significance. The Akaike Information Criterion (AIC) values of these models were lower than the AIC values of multiple Cox regression models adjusted only for age, sex and mean BP or heart rate, indicating better model properties. The AIC values of these models were also lower than the AIC value of the similar multiple Cox regression model that included only demographic parameters and vascular risk factors (AIC: 753.19; Supplementary Figure S2).

## Discussion

**Figure 5:**
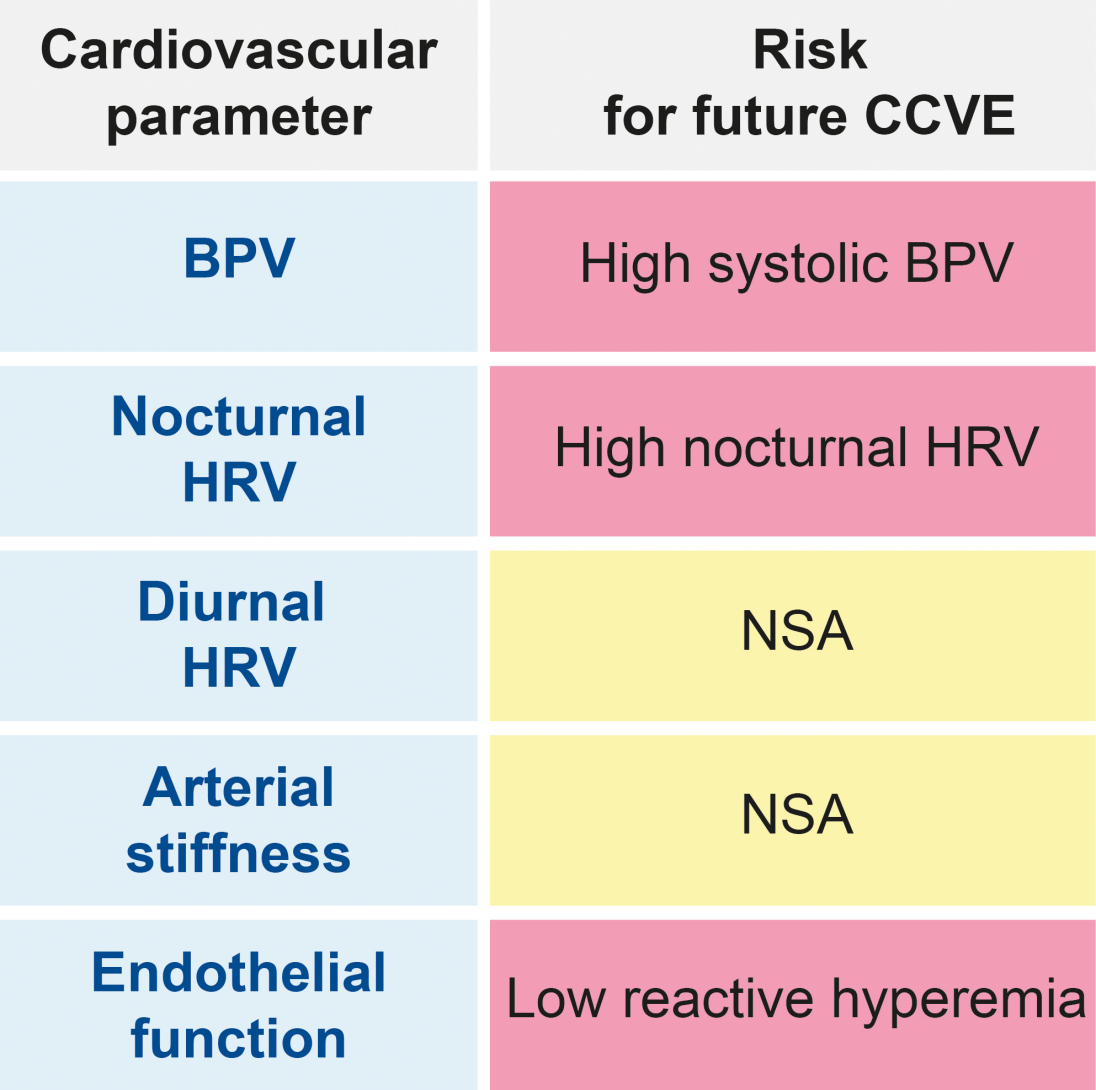
Schematic representation of the associations of cardiovascular parameters at acute stroke with the risk for future CCVE (cerebro-cadriovascular events). NSA, no significant associations.

In this study, we comprehensively describe cardiovascular parameters at acute stroke and explore their ability to predict recurrent CCVE over 3 years (Figure 5). We found that high BPV, high nocturnal HRV and low reactive hyperemia are associated with increased risk of future CCVE in acute stroke patients. Overall, our findings align with established associations between cardiovascular parameters and known vascular risk factors, such as high BPV with sleep-disordered breathing (22) and endothelial dysfunction with hypertension, diabetes and dyslipidemia (23). Prior work demonstrated a reduction in HRV during the nighttime in non-dipping patients with hypertension (24). Similarly, our study revealed a strong positive correlation between high BPV and high nocturnal HRV.

Additionally, we demonstrated the associations between cardiovascular parameters (high BP, BPV, nocturnal HRV) and stroke severity and functional disability. However, it remains uncertain whether these cardiovascular parameters are merely associated with severe stroke or if severe stroke leads to alterations in these parameters. Our study population was skewed towards patients with minor strokes, limiting our ability to predict the actual improvement in stroke outcome associated with these cardiovascular parameters due to floor effects.

Finally, we observed that mean systolic BP, systolic BPV, nocturnal HRV and endothelial dysfunction at acute stroke reflect the risk for future recurrent CCVE in stroke patients, even after adjustment for demographic and cardiovascular risk factors. Indeed, high systolic BP and BPV parameters during the acute stroke phase predict recurrent CCVE, in line with prior research (25). In our cohort, SBP-amplitude was the most indicative parameter with the largest effect size. Although diastolic BP and the other BPV parameters did predict recurrent CCVE in univariate analysis, these associations lost significance after adjustment for demographic and cardiovascular risk factors.

In general, high HRV is typically considered indicative of healthy cardiovascular autonomic function, while low HRV is commonly viewed as a cardiovascular risk factor in the general population without prior cardiovascular events (26). Although most studies concentrated on diurnal HRV (26), Binici et al. (27) demonstrated that low nocturnal HRV, as assessed from time-domain measurements (SDNN), was associated with stroke risk in the general population (*n* = 653). However, within our study population, non-linear HRV-based variables reflecting unpredictable HRV [SD1 and SD2 (27, 28)] during the acute stroke phase were indicative of CCVE risk (28, 29). Our findings accord with the data from HypnoLaus study showing the association of CCVE risk in general population with non-linear HRV measurements reflecting altered acceleration capacity, deceleration capacity and heart rate fragmentation (30). The contrasting patterns of association between nocturnal and diurnal HRV and CCVE risk may be explained by sleep-wake disorders, which are recognized as an independent cardiovascular risk factor (31). For instance, sleep-disordered breathing is common after a stroke and may be accompanied by decreased diurnal HRV and increased nocturnal HRV (32, 33). In turn, the diminished physiological circadian fluctuation of autonomic functions, as reflected by time-domain HRV, was associated with an unfavorable cardiovascular and metabolic profile (24).

Our study did not show an association between diurnal HRV and CCVE risk in stroke patients. While an association of diurnal HRV with CCVE risk was reported in general population (7), our results support a previously reported lack of the association of diurnal HRV with CCVE risk in acute stroke patients (*n* = 308) over 1-year follow-up (9). We assume that this discrepancy might be related to altered mechanisms of resting heart rate regulation in acute stroke.

Furthermore, our study did not confirm the association between arterial stiffness and CCVE risk. Notably, a prior study had reported a link between arterial stiffness and the need for urgent revascularization in patients with a high cardiovascular risk (34). This discrepancy might be related to the difference in outcome measures between the studies. However, we show an association of endothelial dysfunction at acute stroke with future CCVE risk supporting previous findings in patients at high cardiovascular risk (35, 36).

### Strengths and limitations

The strengths of the study include the prospective design with a broad cardiovascular phenotyping. Additionally, we followed stroke patients for CCVEs during a period of 3-years and compared the fit of Cox regression models predicting CCVE risk.

The present study has several limitations. First, sample size and number of events are small for a robust evaluation of a large set of parameters. Second, the large number of exploratory analyses, without control for multiplicity, limits the confidence around key findings, and larger cohort studies are needed to evaluate their applicability.

## Conclusions

In summary, we comprehensively describe cardiovascular parameters (BPV, nocturnal and diurnal HRV, endothelial function and arterial stiffness) in patients with a minor stroke. The risk for future CCVE was associated with high systolic BPV, high and unpredictable non-linear nocturnal HRV and low reactive hyperemia at acute stroke. We propose that phenotyping based on cardiovascular parameters could expand the current knowledge about the prediction of cardiovascular risk.

## Data Availability

The raw data supporting the conclusions of this article will be made available by the authors, without undue reservation.

## Glossary

ApEn, approximate entropy; AI, augmentation index; AI75, augmentation index normalized for 75 bpm; AIC, Akaike Information Criterion; ARV, average real variability; AS, arterial stiffness; BPV, blood pressure variability; CCVE, cerebro-cardiovascular events; CV, coefficient of variation; D, diurnal; DBP, diastolic blood pressure; DFA1, detrended fluctuation analysis component *α*1; DFA2, detrended fluctuation analysis component *α*2; EF, endothelial function; FRHI, Framingham Reactive Hyperemia Index; HFnu, normalized high frequency power; HF, high frequency power; HR, heart rate; HRV, heart rate variability; LFnu, normalized low frequency power; HFnu, normalized high frequency power; LF, low frequency power; MAD, mean absolute deviation; mps, months post-stroke; N, nocturnal; pNN50, percentage of adjacent NN intervals that differ from each other by more than 50 ms; RHI, reactive hyperemia index; RMSSD, root mean square of successive differences of successive RR intervals; SBP, systolic blood pressure; SD, standard deviation; SD1, SD1 from Poincaré plot; SD2, SD2 from Poincaré plot; SDNN, standard deviation of normal-to-normal beats; SDSD, standard deviation of successive RR interval differences; SV, successive variation; TI, triangular index; TIA, transient ischemic attack; TINN, triangular interpolation of NN interval histogram; VLF, very low frequency power.
