# Supplementary material for: Blood pressure variability, nocturnal heart rate variability and endothelial function predict recurrent cerebro-cardiovascular events following ischemic stroke
Source: Front Cardiovasc Med. 2023 Nov 16;10:1288109. doi: 10.3389/fcvm.2023.1288109 (PMC10687449; doi:10.3389/fcvm.2023.1288109)
Supplement: Supplementary file 1 [file Datasheet1.pdf]

## Supplementary material

**Supplementary material 1**. The assessment of cardiovascular parameters.

*Blood pressure*

Mean BP and BPV were assessed based on consecutive in-hospital and outpatient BP measurements conducted within 1-4 days after hospital admission (within clinical routine and study-specific assessments). For the measurements conducted within 1-4 days after hospital admission, we used both the in-hospital BP measurements within clinical routine (frequency is determined by clinical routine protocol dependent on stroke severity) and study-specific ambulatory BP measurements. Ambulatory BP measurements were performed using a boso Medicus PC 2 device (Bosch + Sohn, Germany). The patients were instructed to perform 3 consecutive measurements in the sitting position (back and arm supported) (1) at 2-minute intervals 3 times per day: morning, day and evening; after 3-5 minutes of rest in a quiet room. Only the patients with at least 12 available BP measurements were included in the analysis.

We calculated mean systolic and diastolic blood pressure as well as the characteristics of systolic and diastolic BPV using RStudio v1.3. These BPV characteristics estimated the deviation from a central point ($\text{standard deviation (SD)} = \sqrt{\frac{\sum_{i=1}^{n}(x_i - x_{mean})^2}{n-1}}$, $\text{coefficient of variation (CV)} = \left(\frac{SD}{x_{mean}}\right) * 100$, $\text{mean absolute deviation (MAD)} = \frac{\sum_{i=1}^{n}|x_i - x_{mean}|}{n}$, $\text{variance} = \frac{\sum_{i=1}^{n}(x_i - x_{mean})^2}{n}$), successive deviation ($\text{successive variation (SV)} = \sqrt{\frac{\sum_{i=1}^{n}(x_{i+1} - x_i)^2}{n-1}}$, $\text{average real variability (ARV)} = \frac{\sum_{i=1}^{n-1}|x_{i+1} - x_i|}{n-1}$) and range ($\text{amplitude} = x_{max} - x_{min}$) (2,3).

*Nocturnal heart rate*

Mean nocturnal HR and HRV were assessed based on the available pulse rate data from the nocturnal respiratory polygraphy performed within 7 days after admission. We used the whole respiratory polygraphy recording to calculate HRV without differentiation of potential wake or sleep time since no differences in HRV parameters between different sleep stages and wakefulness were reported in the previous study in acute and subacute stroke patients (4).

Nocturnal HRV was calculated using HRVTool, an Open-Source Matlab Toolbox for Analyzing Heart Rate Variability, by detecting heart beats based on pulse-wave form (5). HRV-parameters were represented in 3 main domains: the time domain was represented by HRV based on relative RR intervals (rrHRV), standard deviation of normal-to-normal beats (SDNN), standard deviation of successive differences (SDSD), triangular index (TI), root mean square of successive differences of successive RR intervals (RMSSD), and the percentage of adjacent NN intervals that differ from each other by more than 50 ms (pNN50). The frequency domain was represented by normalized low frequency (nuLf) power, normalized high frequency (nuHF) power, nuLF/nuHf ratio, low frequency (LF) power, high frequency (HF) power and very low frequency (VLF) power. The quantification of frequency domain HRV was based on spectral analysis of a sequence. Lastly, non-linear measurements were represented by SD1 and SD2 from Poincaré plots, SD1/SD2 ratio, detrended fluctuation analysis α1 (DFA1; reflects short-term fluctuations (6); higher values reflect sympathetic modulation (7)), detrended fluctuation analysis α2 (DFA2; reflects long-term fluctuations (6); higher values reflect sympathetic modulation (7)) and approximate entropy (ApEn).

*EndoPAT: diurnal heart rate, endothelial function and arterial stiffness*

Approximately 20% of the patients were randomly selected for additional assessments of diurnal HRV, endothelial function and arterial stiffness with digital plethysmography (EndoPAT2000-device; Itamar Medical Ltd., Caesarea, Israel). The assessment was performed within the first week after hospital admission according to the instructions from the manufacturer.

The participants were instructed to fast and to refrain from smoking, alcohol- and xanthine-containing beverages for 12 hours prior to the assessment of cardiovascular parameters. The measurement of cardiovascular characteristics was performed in the morning (between 08:00 and 11:00). The participants were in supine position for a minimum of 20 minutes before measurements in a quiet room (temperature 21-24°C) and stayed motionless during the entire measurement period.

Endothelial function, arterial stiffness and diurnal heart rate (mean and HRV) were calculated using the EndoPAT software package version 3.5.3 and were represented as follows: 1) mean HR; 2) time domain of HRV – SDNN, RMSSD, pNN50 and TI; 3) frequency domain of HRV – LF power (LF), HF power (HF) and LF/HF; 4) endothelial function – reactive hyperemia index (RHI) and Framingham reactive hyperemia index (FRHI); 5) arterial stiffness – augmentation index and augmentation index corrected for a heart rate of 75 bpm (AI75).

**Supplementary material 2**. The assessment of cerebro-cardiovascular events.

The main outcome of the study was a composite of fatal and non-fatal recurrent CCVE that included ischemic or hemorrhagic stroke, TIA, myocardial infarction, unplanned hospitalization for unstable angina or heart failure and urgent revascularization. CCVE were assessed by a research fellow or a study nurse in a structured telephone interview over a 3-

year (1095-day period) follow-up after study inclusion. The patients' general practitioner/attending physician was contacted to collect the endpoint if neither patient nor relative was answering or was unable to provide requested information. Death was ascertained by patients' relatives, general practitioners or by an automatic query sent from the hospitals patient management system to the Central Compensation Office that registers all deaths of Swiss citizens or individuals working in Switzerland. If a patient did not have recurrent CCVE and died from a non-CCVE event, the date of death was considered the date of the last contact. If a patient had multiple recurrent CCVE, the earliest recurrent CCVE determined the event-free interval within the investigated time period (1-1095 days).

**Supplementary table 1**. Physiological characteristics of the investigated cardiovascular parameters regarding cerebro-cardiovascular risk.

| Cardiovascular parameter | **Physiological characteristics** |
|---|---|
| Mean SBP and mean DBP | SBP and DBP are well-investigated cardiovascular parameters reflecting cardiac output and vascular resistance (8). The levels of blood pressure are defined by the interplay of multiple factors, such as autonomic tone, endocrine factors, endothelial functioning, arterial stiffness and activity levels (9). With high levels of BP reflecting CCVE risk, the recent meta-analysis suggested that reducing BP levels significantly reduces the risk of cardiovascular disease and all-cause mortality (10). |
| Systolic and diastolic BPV | BPV reflects the fluctuations in BP during the investigated period of time. BPV can be described as the deviation from a central point (mean or median), successive deviation and range (2,3). High values of these parameters indicate high BPV. The recent study suggested that high variability in blood pressure is associated with high CCVE risk in general population (n=42,482) (11). |
| Mean HR | HR is known as a predictor of CCVE risk. Resting heart rate shows U-shaped associations with CCVE risk (12). Night-time HR seems to have better predictive ability regarding CCVE risk compared to daytime and 24-h HR (13). |
| HRV | HRV reflects the fluctuations in HR during the investigated period of time and can be characterized by 3 groups of parameters as specified below (14). |
| Time domain of HRV | These parameters (for example, SDNN, SDSD, RMSSD, pNN50, TI, TINN) quantify the amount of variability in measurements of the interbeat interval, which is the time period between successive |

| | |
|---|---|
| | heartbeats (14). High values of these parameters indicate high HRV. These parameters could reflect both sympathetic and parasympathetic autonomic functioning (14). Low diurnal HRV with high levels of sympathetic components and lower parasympathetic as well as unpredictable nocturnal HRV was described to be associated with CCVE risk and CCVE risk factors (15–18). |
| Frequency domain of HRV | These parameters (for example, nuLF, nuHF, LF, HF, VLF, LF/HF) quantify signal energy within a specific frequency band (14). The physiological regulation of these parameters is complex and involves circadian, endocrine and autonomic components (14,19). There is no consensus regarding the mechanisms that define these signals, however, current literature overview points to the association of LF power with sympathetic activation, HF power with parasympathetic activation and VLF power with circadian rhythm and sympathetic activation (14,20,21). In a simplified way, LF/HF ratio is supposed to reflect sympatho-vagal balance, however, considering the non-linear relationship between sympathetic and parasympathetic regulation, this paradigm is largely debated (22). |
| Non-linear HRV measurements | These parameters (for example, DFAa1, DFAa2, ApEn, SD1, SD2, SD1/SD2) quantify the unpredictability of a time series: high values of these parameters indicate high HRV (14,23). Little is known about the physiological regulation of these parameters. According to few available studies, higher values of DFA, ApEn and SD2 reflect sympathetic modulation, while SD1 could reflect parasympathetic modulation (7,24,25). |
| Endothelial dysfunction: RHI, FRHI | Reactive hyperemia represents the magnitude of limb reperfusion following a brief period of ischemia induced by arterial occlusion (the ratio of hyperemic pressure to baseline pressure in the measurement and control arms) (26). Compared to RHI, FRHI, uses the natural logarithmic transformation of the RHI ratio, does not |

| | |
|---|---|
| | include the baseline correction factor and utilizes only the readings from 90 to 120 seconds for post occlusion (27). Lower RHI and FRHI indicate low hyperemic response and are associated with CCVE risk factors (26). |
| Arterial stiffness: AI, AI75 | AI is calculated as augmentation pressure divided by pulse pressure ×100 to give a percentage (28). AI75 indicates augmentation index corrected for heart rate at 75 beats per minute. High values of AI and AI75 indicate high arterial stiffness and are associated with CCVE risk factors (29). |

Abbreviations: ApEn - approximate entropy, AI – augmentation index, AI75 – augmentation index normalized for 75 bpm, DBP – diastolic blood pressure, DFA1 - detrended fluctuation analysis component α1, DFA2 - detrended fluctuation analysis component α1, FRHI – Framingham Reactive Hyperemia Index, HFnu - normalized high frequency power, HF – high frequency power, HR – heart rate, HRV – heart rate variability, LFnu - normalized low frequency power, LF – low frequency power, MAD – mean absolute deviation, pNN50 - percentage of adjacent NN intervals that differ from each other by more than 50 ms, RHI – reactive hyperemia index, RMSSD - root mean square of successive differences of successive RR intervals, SBP – systolic blood pressure, SD1 – SD1 from Poincaré plot, SD2 – SD2 from Poincaré plot, SDNN – standard deviation of normal-to-normal beats, SDSD - standard deviation of successive RR interval differences, TI – triangular index, TINN - triangular interpolation of NN interval histogram, VLF – very low frequency power.

**Supplementary table 2**. Characteristics of participants with specific cardiovascular assessments at acute stroke (overall n=359). There were no significant differences between the populations with specific cardiovascular assessments.

| Parameter | BPV data (n=333) | | HRV data (n=187) | | EndoPAT data (n=105) | | P-value |
|---|---|---|---|---|---|---|---|
| | N | Value | N | Value | N | Value | |
| Age, years | 333 | 66.90 [57.40, 74.60] | 187 | 67.10 [57.55, 74.85] | 105 | 64.70 [54.70, 73.10] | 0.524 |
| Sex | 333 | 122 (36.6%) | 187 | 69 (36.9%) | 105 | 35 (33.3%) | 0.823 |
| TIA | 333 | 42 (12.6%) | 187 | 14 (7.5%) | 105 | 15 (14.3%) | 0.123 |
| TOAST | 333 | | 187 | | 105 | | 0.757 |
| - CE | | 111 (33.3%) | | 63 (33.7%) | | 36 (34.3%) | |
| - LAA | | 81 (24.3%) | | 46 (24.6%) | | 22 (21.0%) | |
| - Other | | 15 (4.5%) | | 8 (4.3%) | | 7 (6.7%) | |
| - SVO | | 23 (6.9%) | | 15 (8.0%) | | 3 (2.9%) | |
| - Unknown | | 103 (30.9%) | | 55 (29.4%) | | 37 (35.2%) | |
| Stroke topography | 333 | | 187 | | 105 | | 0.530 |
| - Supra | | 226 (67.9) | | 131 (70.1) | | 71 (67.6) | |
| - Infra | | 57 (17.1) | | 38 (20.3) | | 16 (15.2) | |
| - Both | | 8 (2.4) | | 4 (2.1) | | 3 (2.9) | |
| - TIA | | 42 (12.6) | | 14 (7.5) | | 15 (14.3) | |
| Wake-up stroke | 333 | 76 (22.8%) | 187 | 41 (21.9%) | 105 | 22 (21.0%) | 0.931 |
| NIHSS at admission, score | 333 | 2.00 [1.00, 5.00] | 187 | 2.00 [1.00, 5.00] | 105 | 2.00 [0.00, 4.00] | 0.307 |
| NIHSS at discharge, score | 333 | 1.00 [0.00, 2.00] | 187 | 1.00 [0.00, 2.00] | 105 | 1.00 [0.00, 2.00] | 0.624 |
| mRS at 3 months post-stroke, score | 260 | 1.00 [0.00, 1.00] | 148 | 1.00 [0.00, 1.00] | 82 | 1.00 [0.00, 2.00] | 0.648 |
| Intravascular stroke treatment | 333 | 93 (27.9%) | 186 | 64 (34.4%) | 104 | 32 (30.8%) | 0.311 |
| Hypertension | 327 | | 185 | | 105 | | 0.752 |
| - No | | 123 (37.6%) | | 68 (36.8%) | | 41 (39.0%) | |
| - Yes, treated | | 168 (51.4%) | | 96 (51.9%) | | 57 (54.3%) | |
| - Yes, untreated | | 36 (11.0%) | | 21 (11.4%) | | 7 (6.7%) | |
| Diabetes | 333 | | 187 | | 105 | | 0.966 |
| - No | | 280 (84.1%) | | 155 (82.9%) | | 90 (85.7%) | |
| - Yes, treated | | 47 (14.1%) | | 29 (15.5%) | | 13 (12.4%) | |
| - Yes, untreated | | 6 (1.8%) | | 3 (1.6%) | | 2 (1.9%) | |
| Dyslipidemia | 319 | | 178 | | 103 | | 0.942 |
| - No | | 133 (41.7%) | | 76 (42.7%) | | 41 (39.8%) | |
| - Yes, treated | | 92 (28.8%) | | 52 (29.2%) | | 34 (33.0%) | |
| - Yes, untreated | | 94 (29.5%) | | 50 (28.1%) | | 28 (27.2%) | |
| BMI, kg/m$^2$ | 332 | 26.25 [23.78, 29.30] | 187 | 26.30 [23.65, 29.35] | 105 | 27.70 [24.70, 30.00] | 0.119 |
| Obesity[1] | 332 | 65 (19.6%) | 187 | 40 (21.4%) | 105 | 27 (25.7%) | 0.402 |
| CAD | 333 | 43 (12.9%) | 187 | 23 (12.3%) | 105 | 11 (10.5%) | 0.819 |
| Heart failure | 333 | 7 (2.1%) | 186 | 6 (3.2%) | 105 | 4 (3.8%) | 0.476 |
| Atrial fibrillation | 327 | 37 (11.3%) | 183 | 16 (8.7%) | 104 | 12 (11.5%) | 0.649 |
| PAD | 332 | 14 (4.2%) | 187 | 10 (5.3%) | 105 | 5 (4.8%) | 0.842 |
| Current smoking | 330 | 85 (25.8%) | 185 | 48 (25.9%) | 105 | 33 (31.4%) | 0.493 |
| BDI | 288 | 4.00 [1.75, 9.00] | 164 | 5.00 [2.00, 10.00] | 92 | 4.00 [1.00, 9.00] | 0.834 |
| Depression | 288 | 69 (24.0%) | 164 | 45 (27.4%) | 92 | 22 (23.9%) | 0.692 |
| AHI | 304 | 7.75 [3.50, 20.60] | 184 | 10.30 [4.80, 22.35] | 97 | 10.00 [4.30, 22.00] | 0.182 |

| | | | | | | | |
|---|---|---|---|---|---|---|---|
| SDB[2] | 304 | 78 (25.7%) | 184 | 51 (27.7%) | 97 | 28 (28.9%) | 0.778 |
| Previous CCVE | 333 | 88 (26.4%) | 187 | 43 (23.0%) | 105 | 24 (22.9%) | 0.633 |
| Future CCVE | 333 | 68 (20.4%) | 187 | 40 (21.4%) | 105 | 22 (21.0%) | 0.963 |

Continuous data is presented as median [interquartile range]. Categorical data is presented as count (% of total). Kruskall-Wallis test was used for continuous variables, Chi-square test was used for categorical variables.
[1]BMI≥30 kg/m$^2$. [2]SDB is defined as AHI≥20/h.
Abbreviations: AHI – apnea-hypopnea index, BDI – Beck Depression Inventory, BMI – body mass index, CAD – coronary artery disease, CCVE – cerebro-cardiovascular event, CE – cardioembolism, DBP – diastolic blood pressure, HRV – heart rate variability, LAAS – large-artery atherosclerosis, mRS – modified Rankin Scale, NIHSS – National Institute of Health Stroke Scale, PAD – peripheral artery disease, SDB – sleep-disordered breathing, SVO – small vessel occlusion, TIA – transient ischemic attack.

**Supplementary table 3**. Description of cardiovascular characteristics at acute stroke.

| Parameter | Value | Range |
|---|---|---|
| Blood pressure (n=67) | | |
| SBP-mean, mmHg | 133.77 [119.14, 146.47] | 99.35-181.91 |
| SBP-SD, mmHg | 12.31 [9.32, 16.45] | 5.43-35.49 |
| SBP-CV, % | 9.38 [7.64, 10.96] | 4.59-35.72 |
| SBP-MAD, mmHg | 9.57 [7.24, 12.86] | 4.50-34.17 |
| SBP-VAR, mmHg | 151.65 [86.80, 270.86] | 29.50-1259.52 |
| SBP-SV, mmHg | 178.17 [123.72, 320.50] | 42.44-1492.54 |
| SBP-ARV, mmHg | 10.40 [9.09, 14.13] | 4.67-35.31 |
| SBP-amplitude, mmHg | 46.00 [35.50, 61.50] | 22.00-141.00 |
| DBP-mean, mmHg | 77.22 [68.96, 84.74] | 51.71-97.43 |
| DBP-SD, mmHg | 9.13 [7.36, 11.52] | 5.02-22.08 |
| DBP-CV, % | 12.65 [9.51, 14.92] | 5.87-23.49 |
| DBP-MAD, mmHg | 7.00 [5.73, 8.54] | 3.77-15.71 |
| DBP-VAR, mmHg | 83.30 [54.18, 132.60] | 25.24-487.38 |
| DBP-amplitude, mmHg | 35.00 [27.00, 44.00] | 18.00-91.00 |
| DBP-SV, mmHg | 122.27 [79.36, 193.23] | 37.75-1370.62 |
| DBP-ARV, mmHg | 8.57 [7.02, 10.65] | 4.65-29.23 |
| Nocturnal heart rate (n=34) | | |
| Mean HR, bpm | 65.36 [57.96, 72.42] | 43.77-83.22 |
| rrHRV, ms | 2.69 [2.27, 3.75] | 1.52-13.63 |
| SDNN, ms | 105.95 [86.35, 130.05] | 59.73-260.49 |
| SDSD, ms | 41.39 [31.24, 54.46] | 24.58-167.34 |
| RMSSD, ms | 41.39 [31.25, 54.47] | 24.58-167.34 |
| pNN50, % | 7.88 [4.25, 18.50] | 1.74-71.54 |
| TI | 12.55 [10.28, 15.95] | 3.59-30.32 |
| TINN | 0.20 [0.15, 0.27] | 0.02-0.54 |
| LFnu, % | 66.59 [58.94, 72.43] | 55.13-81.07 |
| HFnu, % | 33.41 [27.57, 41.06] | 18.93-44.87 |
| LF/HF, ms2 | 1.99 [1.44, 2.63] | 1.23-4.28 |
| VLF, ms2 | 1.63 [1.30, 2.29] | 0.62-2.90 |
| LF, ms2 | 0.61 [0.52, 0.81] | 0.11-1.27 |
| HF, ms2 | 0.29 [0.23, 0.44] | 0.06-1.04 |
| DFA1 | 0.90 [0.80, 0.97] | 0.72-1.16 |
| DFA2 | 0.81 [0.74, 0.91] | 0.61-1.00 |
| ApEn,ms | 0.87 [0.70, 1.25] | 0.49-1.98 |
| SD1, ms | 0.34 [0.22, 0.68] | 0.07-70.97 |
| SD2, ms | 0.41 [0.28, 0.72] | 0.13-70.98 |
| SD1/SD2 | 0.87 [0.76, 0.92] | 0.54-1.00 |
| Diurnal heart rate (n=66) | | |
| Mean HR, bpm | 62.74 [56.64, 69.26] | 37.69-91.46 |
| pNN50, % | 0.02 [0.00, 0.06] | 0.00-0.40 |
| RMSSD, ms | 27.96 [19.72, 42.74] | 9.37-259.44 |

| | | |
|---|---|---|
| SDNN, ms | 40.42 [30.68, 61.11] | 14.36-182.63 |
| TI | 10.22 [8.37, 13.13] | 4.19-36.00 |
| LF, ms2 | 111.17 [81.62, 161.98] | 33.96-283.21 |
| HF, ms2 | 98.10 [68.04, 159.64] | 12.64-284.43 |
| LF/HF | 1.09 [0.59, 2.46] | 0.20-9.34 |
| Endothelial dysfunction (n=66) | | |
| RHI | 2.00 [1.66, 2.31] | 1.03-3.20 |
| FRHI | 0.48 [0.22, 0.63] | -0.36-1.12 |
| Arterial stiffness (n=66) | | |
| AI, % | 21.63 [8.45, 35.40] | -23.96-87.27 |
| AI75, % | 16.08 [4.15, 25.40] | -38.33-77.27 |

*p<0.05, Wilcoxon signed-rank test

Abbreviations: ApEn - approximate entropy, AI – augmentation index, AI75 – augmentation index normalized for 75 bpm, ARV – average real variability, bpm – beats per minute, CV – coefficient of variation, DBP – diastolic blood pressure, DFA1 - detrended fluctuation analysis component α1, DFA2 - detrended fluctuation analysis component α1, FRHI – Framingham Reactive Hyperemia Index, HFnu - normalized high frequency power, HF – high frequency power, HR – heart rate, HRV – heart rate variability, LFnu - normalized low frequency power, LF – low frequency power, MAD – mean absolute deviation, pNN50 - percentage of adjacent NN intervals that differ from each other by more than 50 ms, RHI – reactive hyperemia index, RMSSD - root mean square of successive differences of successive RR intervals, SBP – systolic blood pressure, SD – standard deviation, SD1 – SD1 from Poincaré plot, SD2 – SD2 from Poincaré plot, SDNN – standard deviation of normal-to-normal beats, SDSD - standard deviation of successive RR interval differences, SV – successive variation, TI – triangular index, TINN - triangular interpolation of NN interval histogram, VLF – very low frequency power.

**Supplementary table 4.** Significant (p<0.05) associations between cardiovascular parameters at acute stroke with demographic characteristics, stroke characteristics and comorbidities according to correlation analysis followed by multiple linear regression analysis (both approaches lead to significance). Multiple linear regression was adjusted for age and sex.

| Dependent | Independent | N | Sperman's Rho | P-value | Estimate | P-value |
|---|---|---|---|---|---|---|
| AI, % | Heart failure | 105 | -0.242 | 0.013 | -19.753 | 0.028 |
| DBP-amplitude, mmHg | AHI, /h | 304 | 0.201 | <0.001 | 0.106 | 0.048 |
| DBP-amplitude, mmHg | NIHSS at admission, score | 333 | 0.139 | 0.011 | 0.433 | 0.020 |
| DBP-amplitude, mmHg | NIHSS at discharge, score | 333 | 0.206 | <0.001 | 0.748 | 0.047 |
| DBP-amplitude, mmHg | TIA | 333 | -0.141 | 0.010 | -6.503 | 0.006 |
| DBP-ARV, mmHg | AHI, /h | 304 | 0.168 | 0.003 | 0.026 | 0.027 |
| DBP-ARV, mmHg | BDI, score | 288 | 0.117 | 0.047 | 0.089 | 0.004 |
| DBP-ARV, mmHg | NIHSS at discharge, score | 333 | 0.229 | <0.001 | 0.213 | 0.012 |
| DBP-ARV, mmHg | TIA | 333 | -0.115 | 0.035 | -1.328 | 0.013 |
| DBP-CV, % | Diabetes | 333 | 0.155 | 0.005 | 1.477 | 0.031 |
| DBP-CV, % | NIHSS at admission, score | 333 | 0.123 | 0.024 | 0.127 | 0.031 |
| DBP-MAD, mmHg | AHI, /h | 304 | 0.195 | 0.001 | 0.019 | 0.032 |
| DBP-MAD, mmHg | NIHSS at admission, score | 333 | 0.118 | 0.031 | 0.062 | 0.044 |
| DBP-mean, mmHg | Current smoking | 330 | -0.114 | 0.039 | -4.576 | 0.001 |
| DBP-mean, mmHg | Diabetes | 333 | -0.154 | 0.005 | -5.052 | 0.002 |
| DBP-mean, mmHg | PAD | 332 | -0.145 | 0.008 | -8.674 | 0.004 |
| DBP-mean, mmHg | Wake-up stroke | 333 | 0.205 | <0.001 | 4.948 | 0.001 |
| DBP-SD, mmHg | AHI, /h | 304 | 0.205 | <0.001 | 0.028 | 0.016 |
| DBP-SD, mmHg | TIA | 333 | -0.138 | 0.011 | -1.304 | 0.011 |
| DBP-SV, mmHg | AHI, /h | 304 | 0.184 | 0.001 | 1.132 | 0.029 |
| DBP-SV, mmHg | TIA | 333 | -0.134 | 0.015 | -56.921 | 0.013 |
| DBP-VAR, mmHg | AHI, /h | 304 | 0.205 | <0.001 | 0.621 | 0.031 |
| DFA1 (N), AU | mRS at 3 mps, score | 148 | -0.194 | 0.018 | -0.028 | 0.027 |
| DFA1 (N), AU | NIHSS at discharge, score | 187 | -0.217 | 0.003 | -0.009 | 0.019 |
| FRHI, AU | Diabetes | 105 | -0.22 | 0.024 | -0.206 | 0.016 |
| FRHI, AU | Dyslipidemia | 103 | -0.198 | 0.044 | -0.134 | 0.033 |
| FRHI, AU | Hypertension | 105 | -0.254 | 0.009 | -0.143 | 0.027 |
| HF (N), ms2 | TIA | 187 | -0.191 | 0.009 | -148.624 | 0.021 |
| LF/HF (N), ms2 | NIHSS at discharge, score | 187 | -0.163 | 0.025 | -0.055 | 0.036 |

| | | | | | | |
|---|---|---|---|---|---|---|
| Mean HR (D), bpm | TIA | 105 | 0.229 | 0.019 | 7.321 | 0.016 |
| nHF (N), % | TIA | 187 | -0.149 | 0.042 | -4.513 | 0.046 |
| nLF (N), % | TIA | 187 | 0.149 | 0.042 | 4.513 | 0.046 |
| RHI, AU | Diabetes | 105 | -0.232 | 0.017 | -0.337 | 0.025 |
| RHI, AU | Dyslipidemia | 103 | -0.198 | 0.045 | -0.243 | 0.026 |
| RHI, AU | Hypertension | 105 | -0.241 | 0.013 | -0.255 | 0.024 |
| RHI, AU | TIA | 105 | -0.241 | 0.013 | -0.318 | 0.036 |
| SBP-amplitude, mmHg | mRS at 3 mps, score | 260 | 0.166 | 0.007 | 4.838 | 0.001 |
| SBP-amplitude, mmHg | NIHSS at admission, score | 333 | 0.217 | <0.001 | 0.711 | 0.009 |
| SBP-amplitude, mmHg | NIHSS at discharge, score | 333 | 0.271 | <0.001 | 2.405 | <0.001 |
| SBP-ARV, mmHg | AHI, /h | 304 | 0.204 | <0.001 | 0.039 | 0.018 |
| SBP-ARV, mmHg | NIHSS at discharge, score | 333 | 0.215 | <0.001 | 0.422 | <0.001 |
| SBP-CV, % | mRS at 3 mps, score | 260 | 0.153 | 0.013 | 0.557 | 0.024 |
| SBP-CV, % | NIHSS at admission, score | 333 | 0.171 | 0.002 | 0.104 | 0.025 |
| SBP-CV, % | NIHSS at discharge, score | 333 | 0.202 | <0.001 | 0.219 | 0.02 |
| SBP-MAD, mmHg | mRS at 3 mps, score | 260 | 0.129 | 0.038 | 0.596 | 0.041 |
| SBP-MAD, mmHg | NIHSS at discharge, score | 333 | 0.222 | <0.001 | 0.391 | 0.001 |
| SBP-mean, mmHg | Hypertension | 327 | 0.268 | <0.001 | 5.636 | 0.012 |
| SBP-mean, mmHg | NIHSS at discharge, score | 333 | 0.205 | <0.001 | 2.200 | <0.001 |
| SBP-SD, mmHg | mRS at 3 mps, score | 260 | 0.136 | 0.028 | 0.855 | 0.015 |
| SBP-SD, mmHg | NIHSS at admission, score | 333 | 0.176 | 0.001 | 0.146 | 0.029 |
| SBP-SD, mmHg | NIHSS at discharge, score | 333 | 0.243 | <0.001 | 0.538 | <0.001 |
| SBP-SV, mmHg | AHI, /h | 304 | 0.201 | <0.001 | 1.874 | 0.026 |
| SBP-SV, mmHg | NIHSS at discharge, score | 333 | 0.218 | <0.001 | 21.568 | <0.001 |
| SBP-VAR, mmHg | mRS at 3 mps, score | 260 | 0.136 | 0.028 | 30.056 | 0.022 |
| SBP-VAR, mmHg | NIHSS at admission, score | 333 | 0.176 | 0.001 | 5.088 | 0.040 |
| SBP-VAR, mmHg | NIHSS at discharge, score | 333 | 0.243 | <0.001 | 19.046 | <0.001 |
| SD1/SD2 (N), AU | BDI, score | 164 | 0.201 | 0.010 | 0.004 | 0.007 |
| VLF (N), ms2 | Atrial fibrillation | 183 | -0.273 | <0.001 | -0.516 | 0.001 |
| VLF (N), ms2 | mRS at 3 mps, score | 148 | -0.209 | 0.011 | -0.158 | 0.013 |

Abbreviations: AF – atrial fibrillation, AHI – apnea-hypopnea index, AI – augmentation index, ARV – average real variability, bpm – beats per minute, BDI – Beck Depression Inventory, CV – coefficient of variation, DBP – diastolic blood pressure, DFA1 - detrended fluctuation analysis component α1, FRHI – Framingham Reactive Hyperemia Index, MAD – mean absolute deviation, mRS – modified Rankin Scale, mps – months post-stroke, NIHSS – National Institute of Health Stroke Scale, PAD – peripheral artery disease, SBP – systolic blood pressure, SD – standard deviation, SD1 – SD1 from Poincaré plot, SD2 – SD2 from Poincaré plot, SDNN – standard deviation of normal-to-normal beats, SDSD - standard deviation of successive RR interval differences, SV – successive variation, VLF – very low frequency power.

**Supplementary table 5.** Summary of Cox regression models of the association of cardiovascular parameters at acute stroke with the risk for recurrent CCVE

| **Model** | **Univariate** | **Adjusted for age, sex[1]** | **Adjusted1 for age, sex, cardiovascular risk factors[1,2]** |
|---|---|---|---|
| N | BPV: 333 with 68 events<br>HRV: 187 with 40 events<br>EndoPAT: 105 with 22 events | BPV: 333 with 68 events<br>HRV: 187 with 40 events<br>EndoPAT: 105 with 22 events | BPV: 307 with 64 events<br>HRV: 171 with 37 events<br>EndoPAT: 102 with 21 events |
| Systolic blood, pressure (SBP) | | | |
| Mean SBP[1] | Hazard ratio 1.47, CI [1.16; 1.87], p=0.001*, AIC: 736.54 | Hazard ratio 1.37, CI [1.06; 1.78], p=0.016*, AIC: 735.22 | Hazard ratio 1.37, CI [1.04; 1.82], p=0.026*, AIC: 687.30 |
| SBP-SD | Hazard ratio 1.46, CI [1.21; 1.76], p<0.001*, AIC: 732.83 | Hazard ratio 1.35, CI [1.08; 1.69], p=0.009*, AIC: 731.29 | Hazard ratio 1.43, CI [1.12; 1.82], p=0.004*, AIC: 682.01 |
| SBP-CV | Hazard ratio 1.32, CI [1.12; 1.55], p<0.001*, AIC: 738.00 | Hazard ratio 1.32, CI [1.09; 1.60], p=0.004*, AIC: 730.65 | Hazard ratio 1.38, CI [1.13; 1.69], p=0.002*, AIC: 681.27 |
| SBP-variance | Hazard ratio 1.32, CI [1.12; 1.55], p<0.001*, AIC: 737.62 | Hazard ratio 1.23, CI [1.01; 1.50], p=0.038*, AIC: 733.61 | Hazard ratio 1.28, CI [1.04; 1.58], p=0.022*, AIC: 684.84 |
| SPB-MAD | Hazard ratio 1.40, CI [1.17; 1.68], p<0.001*, AIC: 735.36 | Hazard ratio 1.30, CI [1.05; 1.61], p=0.018*, AIC: 732.44 | Hazard ratio 1.36, CI [1.08; 1.72], p=0.009*, AIC: 683.37 |
| SPB-SV | Hazard ratio 1.33, CI [1.12; 1.58], p=0.001*, AIC: 738.18 | Hazard ratio 1.16, CI [0.93; 1.45], p=0.182, AIC: 735.58 | Hazard ratio 1.19, CI [0.94; 1.51], p=0.157, AIC: 687.46 |

| | | | |
|---|---|---|---|
| SPB-ARV | Hazard ratio 1.46, CI [1.21; 1.76], p<0.001*, AIC: 733.2 | Hazard ratio 1.30, CI [1.03; 1.64], p=0.030*, AIC: 732.91 | Hazard ratio 1.31, CI [1.02; 1.69], p=0.034*, AIC: 685.17 |
| SPB-amplitude | Hazard ratio 1.51, CI [1.26; 1.81], p<0.001*, AIC: 729.79 | Hazard ratio 1.43, CI [1.14; 1.80], p=0.002*, AIC: 729.02 | Hazard ratio 1.50, CI [1.18; 1.90], p<0.001*, AIC: 679.81 |
| Diastolic blood, pressure (DBP) | | | |
| Mean DBP[1] | Hazard ratio 1.09, CI [0.86; 1.4], p=0.473, AIC: 745.96 | Hazard ratio 1.10, CI [0.85; 1.44], p=0.465, AIC: 740.40 | Hazard ratio 1.09, CI [0.83; 1.43], p=0.548, AIC: 691.89 |
| DBP-SD | Hazard ratio 1.35, CI [1.11; 1.63], p=0.003*, AIC: 738.59 | Hazard ratio 1.27, CI [1.04; 1.55], p=0.022*, AIC: 737.67 | Hazard ratio 1.24, CI [0.99; 1.54], p=0.060, AIC: 690.63 |
| DBP-CV | Hazard ratio 1.29, CI [1.05; 1.59], p=0.014*, AIC: 741.09 | Hazard ratio 1.31, CI [1.04; 1.64], p=0.021*, AIC: 737.61 | Hazard ratio 1.27, CI [0.99; 1.63], p=0.056, AIC: 690.50 |
| DBP-variance | Hazard ratio 1.25, CI [1.05; 1.48], p=0.011*, AIC: 741.29 | Hazard ratio 1.18, CI [0.99; 1.41], p=0.068, AIC: 739.57 | Hazard ratio 1.16, CI [0.95; 1.41], p=0.142, AIC: 692.00 |
| DBP-MAD | Hazard ratio 1.31, CI [1.09; 1.58], p=0.005*, AIC: 739.67 | Hazard ratio 1.23, CI [1.01; 1.5], p=0.038*, AIC: 738.58 | Hazard ratio 1.21, CI [0.98; 1.51], p=0.080, AIC: 691.08 |
| DBP-SV | Hazard ratio 1.14, CI [0.95; 1.35], p=0.154, AIC: 744.78 | Hazard ratio 1.07, CI [0.88; 1.29], p=0.494, AIC: 741.97 | Hazard ratio 1.03, CI [0.83; 1.28], p=0.807, AIC: 693.83 |
| DBP-ARV | Hazard ratio 1.24, CI [1.02; 1.50], p=0.032*, AIC: 742.43 | Hazard ratio 1.15, CI [0.94; 1.42], p=0.179, AIC: 740.73 | Hazard ratio 1.10, CI [0.88; 1.38], p=0.402, AIC: 693.22 |
| DBP-amplitude | Hazard ratio 1.32, CI [1.08; 1.60], p=0.006*, AIC: 739.83 | Hazard ratio 1.28, CI [1.04; 1.58], p=0.021*, AIC: 737.58 | Hazard ratio 1.25, CI [1.00; 1.57], p=0.050, AIC: 690.34 |
| Nocturnal heart rate (HR) | | | |
| Mean HR[1] | Hazard ratio 1.04, CI [0.77; 1.42], p=0.780, AIC: 391.64 | Hazard ratio 1.05, CI [0.76; 1.44], p=0.765, AIC: 390.9 | Hazard ratio 1.01, CI [0.73; 1.41], p=0.948, AIC: 357.44 |
| rrHRV | Hazard ratio 1.05, CI [0.76; 1.45], p=0.763, AIC: 391.63 | Hazard ratio 1.14, CI [0.83; 1.56], p=0.410, AIC: 392.28 | Hazard ratio 1.06, CI [0.76; 1.49], p=0.719, AIC: 359.32 |
| SDNN | Hazard ratio 1.28, CI [1.01; 1.62], p=0.043*, AIC: 388.41 | Hazard ratio 1.28, CI [0.99; 1.64], p=0.055, AIC: 389.79 | Hazard ratio 1.29, CI [1.00; 1.66], p=0.052, AIC: 356.21 |
| SDSD | Hazard ratio 1.23, CI [0.95; 1.60], p=0.114, AIC: 389.57 | Hazard ratio 1.24, CI [0.96; 1.62], p=0.105, AIC: 390.63 | Hazard ratio 1.18, CI [0.90; 1.56], p=0.240, AIC: 358.19 |
| RMSSD | Hazard ratio 1.27, CI [0.98; 1.65], p=0.071, AIC: 388.99 | Hazard ratio 1.28, CI [0.98; 1.67], p=0.067, AIC: 390.06 | Hazard ratio 1.22, CI [0.92; 1.60], p=0.166, AIC: 357.73 |
| pNN50 | Hazard ratio 1.05, CI [0.77; 1.44], p=0.756, AIC: 391.63 | Hazard ratio 1.15, CI [0.84; 1.58], p=0.396, AIC: 392.22 | Hazard ratio 1.07, CI [0.76; 1.50], p=0.709, AIC: 359.31 |
| TINN | Hazard ratio 0.84, CI [0.60; 1.18], p=0.306, AIC: 390.59 | Hazard ratio 0.91, CI [0.65; 1.28], p=0.581, AIC: 392.58 | Hazard ratio 0.95, CI [0.69; 1.32], p=0.771, AIC: 359.36 |
| nuLF | Hazard ratio 0.87, CI [0.63; 1.20], p=0.402, AIC: 391.02 | Hazard ratio 0.87, CI [0.63; 1.20], p=0.398, AIC: 392.19 | Hazard ratio 0.89, CI [0.63; 1.24], p=0.475, AIC: 358.94 |
| nuHF | Hazard ratio 1.15, CI [0.83; 1.58], p=0.402, AIC: 391.02 | Hazard ratio 1.15, CI [0.83; 1.60], p=0.398, AIC: 392.19 | Hazard ratio 1.13, CI [0.81; 1.58], p=0.475, AIC: 358.94 |
| LF/HF | Hazard ratio 0.97, CI [0.70; 1.34], p=0.855, AIC: 391.69 | Hazard ratio 0.96, CI [0.70; 1.33], p=0.829, AIC: 392.85 | Hazard ratio 1.00, CI [0.71; 1.40], p=0.984, AIC: 359.44 |
| VLF | Hazard ratio 0.96, CI [0.70; 1.31], p=0.783, AIC: 391.64 | Hazard ratio 1.02, CI [0.74; 1.41], p=0.889, AIC: 392.88 | Hazard ratio 1.12, CI [0.79; 1.59], p=0.507, AIC: 359.01 |
| LF | Hazard ratio 1.05, CI [0.76; 1.44], p=0.776, AIC: 391.64 | Hazard ratio 1.00, CI [0.73; 1.36], p=0.980, AIC: 392.9 | Hazard ratio 0.98, CI [0.70; 1.37], p=0.922, AIC: 359.43 |
| HF | Hazard ratio 1.27, CI [0.94; 1.70], p=0.116, AIC: 389.49 | Hazard ratio 1.25, CI [0.91; 1.71], p=0.173, AIC: 391.15 | Hazard ratio 1.22, CI [0.88; 1.69], p=0.235, AIC: 358.1 |
| DFA1 | Hazard ratio 0.76, CI [0.53; 1.09], p=0.132, AIC: 389.27 | Hazard ratio 0.82, CI [0.57; 1.20], p=0.306, AIC: 391.8 | Hazard ratio 0.86, CI [0.61; 1.23], p=0.420, AIC: 358.77 |
| DFA2 | Hazard ratio 0.87, CI [0.62; 1.20], p=0.387, AIC: 390.96 | Hazard ratio 0.98, CI [0.70; 1.38], p=0.913, AIC: 392.89 | Hazard ratio 1.02, CI [0.72; 1.45], p=0.910, AIC: 359.43 |
| ApEn | Hazard ratio 0.80, CI [0.57; 1.12], p=0.197, AIC: 389.99 | Hazard ratio 0.87, CI [0.62; 1.22], p=0.411, AIC: 392.2 | Hazard ratio 0.76, CI [0.53; 1.09], p=0.139, AIC: 357.15 |
| SD1 | Hazard ratio 1.16, CI [0.99; 1.37], p=0.063, AIC: 389.64 | Hazard ratio 1.22, CI [1.03; 1.45], p=0.019*, AIC: 389.82 | Hazard ratio 1.28, CI [1.07; 1.52], p=0.006*, AIC: 355.34 |
| SD2 | Hazard ratio 1.17, CI [0.99; 1.37], p=0.062, AIC: 389.63 | Hazard ratio 1.22, CI [1.03; 1.45], p=0.019*, AIC: 389.81 | Hazard ratio 1.28, CI [1.07; 1.52], p=0.006*, AIC: 355.33 |
| SD1/SD2 | Hazard ratio 1.19, CI [0.84; 1.69], p=0.331, AIC: 390.72 | Hazard ratio 1.22, CI [0.85; 1.74], p=0.275, AIC: 391.64 | Hazard ratio 1.14, CI [0.78; 1.66], p=0.499, AIC: 358.98 |
| Diurnal heart rate | | | |

| | | | |
|---|---|---|---|
| Mean HR, bpm | Hazard ratio 0.82, CI [0.52; 1.28], p=0.380, AIC: 191.26 | Hazard ratio 0.82, CI [0.51; 1.33], p=0.424, AIC: 188.58 | Hazard ratio 0.89, CI [0.54; 1.47], p=0.639, AIC: 186.09 |
| pNN50 | Hazard ratio 0.72, CI [0.38; 1.37], p=0.320, AIC: 190.83 | Hazard ratio 0.75, CI [0.36; 1.58], p=0.452, AIC: 189.9 | Hazard ratio 0.75, CI [0.38; 1.50], p=0.420, AIC: 187.3 |
| RMSSD | Hazard ratio 0.82, CI [0.35; 1.93], p=0.658, AIC: 191.83 | Hazard ratio 0.90, CI [0.35; 2.34], p=0.834, AIC: 190.53 | Hazard ratio 0.83, CI [0.32; 2.10], p=0.688, AIC: 187.9 |
| SDNN | Hazard ratio 0.82, CI [0.46; 1.44], p=0.480, AIC: 191.5 | Hazard ratio 0.91, CI [0.48; 1.74], p=0.783, AIC: 190.5 | Hazard ratio 0.92, CI [0.49; 1.72], p=0.787, AIC: 188.01 |
| TI | Hazard ratio 0.92, CI [0.57; 1.48], p=0.725, AIC: 191.93 | Hazard ratio 1.02, CI [0.59; 1.75], p=0.951, AIC: 190.57 | Hazard ratio 1.06, CI [0.62; 1.82], p=0.834, AIC: 188.04 |
| LF | Hazard ratio 0.66, CI [0.40; 1.11], p=0.115, AIC: 189.16 | Hazard ratio 0.78, CI [0.44; 1.39], p=0.397, AIC: 189.81 | Hazard ratio 0.81, CI [0.45; 1.44], p=0.469, AIC: 187.53 |
| HF | Hazard ratio 0.82, CI [0.51; 1.32], p=0.422, AIC: 191.37 | Hazard ratio 0.74, CI [0.45; 1.21], p=0.229, AIC: 189.04 | Hazard ratio 0.65, CI [0.39; 1.10], p=0.108, AIC: 185.29 |
| LF/HF | Hazard ratio 1.12, CI [0.77; 1.65], p=0.550, AIC: 191.72 | Hazard ratio 1.16, CI [0.84; 1.60], p=0.376, AIC: 189.89 | Hazard ratio 1.29, CI [0.91; 1.83], p=0.148, AIC: 186.3 |
| Endothelial function | | | |
| RHI | Hazard ratio 0.79, CI [0.50; 1.26], p=0.322, AIC: 191.01 | Hazard ratio 0.82, CI [0.52; 1.29], p=0.394, AIC: 188.5 | Hazard ratio 0.92, CI [0.56; 1.51], p=0.745, AIC: 186.2 |
| FRHI | Hazard ratio 0.64, CI [0.41; 0.98], p=0.042*, AIC: 187.91 | Hazard ratio 0.63, CI [0.40; 1.00], p=0.048*, AIC: 185.28 | Hazard ratio 0.68, CI [0.41; 1.11], p=0.124, AIC: 183.9 |
| Arterial stiffness | | | |
| AI | Hazard ratio 1.14, CI [0.74; 1.77], p=0.545, AIC: 191.69 | Hazard ratio 0.90, CI [0.55; 1.48], p=0.669, AIC: 189.06 | Hazard ratio 0.91, CI [0.54; 1.53], p=0.710, AIC: 186.17 |
| AI75 | Hazard ratio 1.06, CI [0.68; 1.65], p=0.785, AIC: 191.98 | Hazard ratio 0.81, CI [0.50; 1.32], p=0.396, AIC: 188.55 | Hazard ratio 0.86, CI [0.52; 1.42], p=0.550, AIC: 185.95 |

*p<0.05

Cardiovascular parameters were represented as sample-based z-scores.

Hazards ratio and confidence interval (CI) are reported in units per standard deviation.

[1]Multivariate models including blood pressure variability or heart rate variability were respectively adjusted for mean SBP, mean DBP, mean nocturnal HR or mean diurnal HR.

[2]Hypertension, diabetes, dyslipidemia, atrial fibrillation, current smoking.

**Supplementary figure 1.** Correlation plot of the associations between cardiovascular parameters at acute stroke.

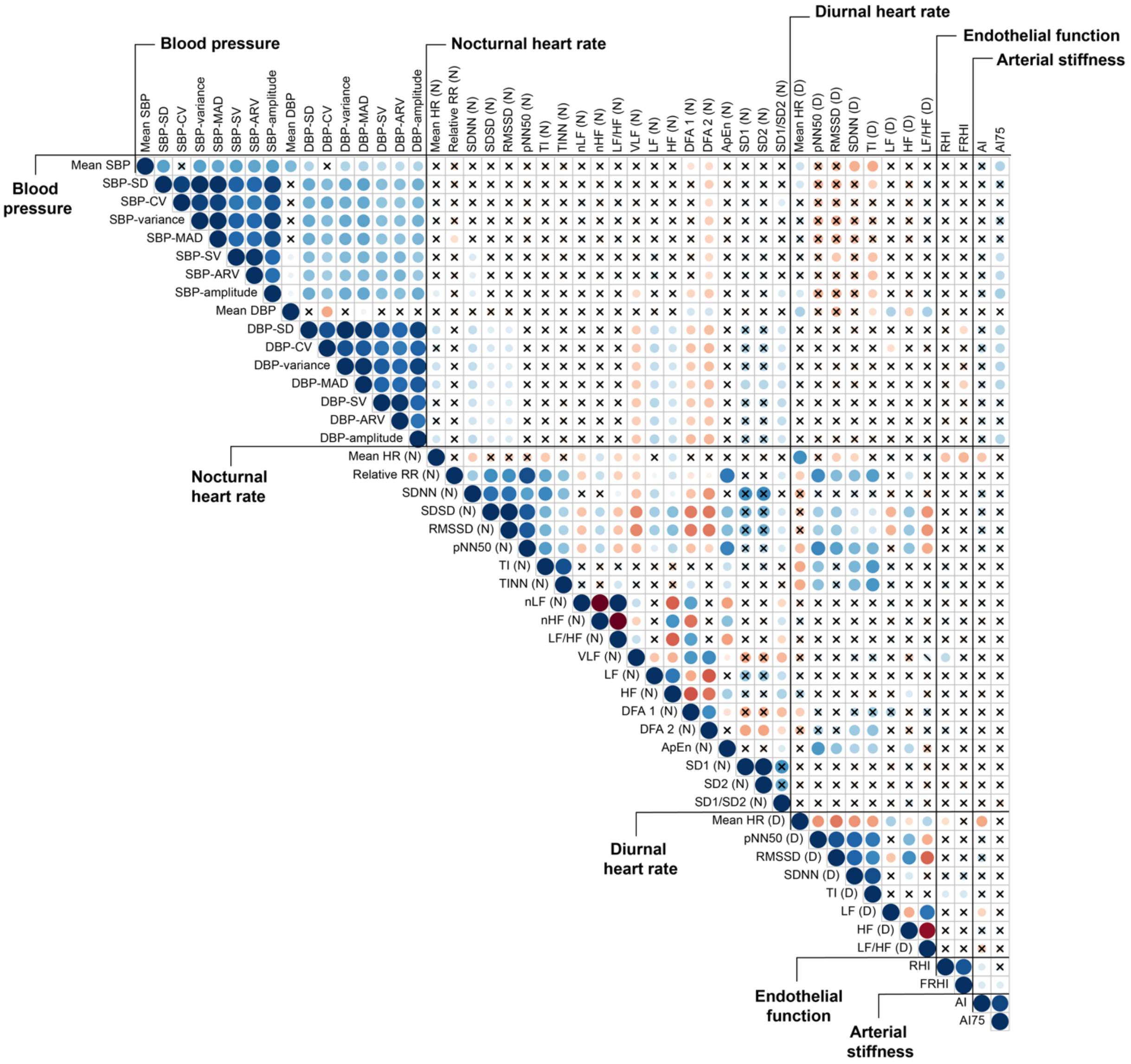


Significant (p<0.05) associations according to Spearman correlation test are shown as filled circles. Crosses mark the insignificant (p≥0.050) associations. Blank squares mark the associations that remained significant in multiple regression models after adjustment for age and sex.

Abbreviations: ApEn - approximate entropy, AI – augmentation index, AI75 – augmentation index normalized for 75 bpm, ARV – average real variability, AS – arterial stiffness, CV – coefficient of variation, DBP – diastolic blood pressure, DFA1 - detrended fluctuation analysis component α1, DFA2 - detrended fluctuation analysis component α2, EF – endothelial function, FRHI – Framingham Reactive Hyperemia Index, HFnu - normalized high frequency power, HF – high frequency power, HR – heart rate, HRV – heart rate variability, LFnu - normalized low frequency power, LF – low frequency power, MAD – mean absolute deviation, pNN50 - percentage of adjacent NN intervals that differ from each other by more than 50 ms, RHI – reactive hyperemia index, RMSSD - root mean square of successive differences of successive RR intervals, SBP – systolic blood pressure, SD – standard deviation, SD1 – SD1 from Poincaré plot, SD2 – SD2 from Poincaré plot, SDNN – standard deviation of normal-to-normal beats, SDSD - standard deviation of successive RR interval differences, SV – successive variation, TI – triangular index, TINN - triangular interpolation of NN interval histogram, VLF – very low frequency power.

**Supplementary figure 2.** Coefficient plot of Cox multiple regression model of the association between cardiovascular risk factors and cerebro-cardiovascular events (CCVE).

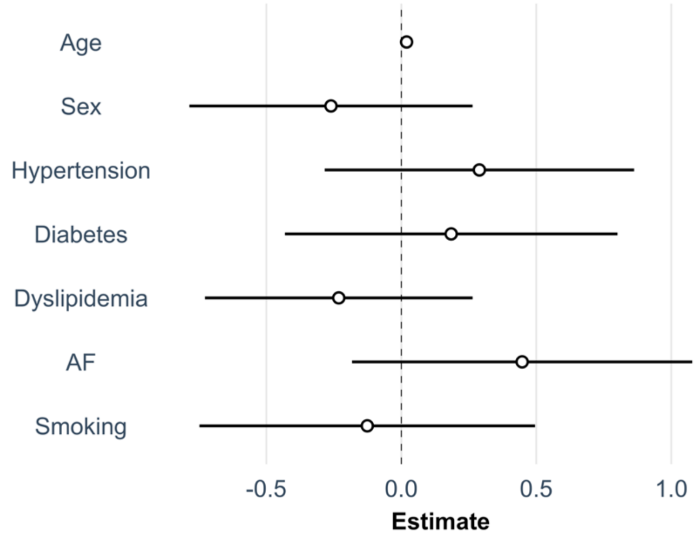


Abbreviations: AF – atrial fibrillation.

## References for supplementary material


1. Williams B, Mancia G, Spiering W, Rosei EA, Azizi M, Burnier M, et al. 2018 Guidelines for the Management of Arterial Hypertension: The Task Force for the Management of Arterial Hypertension of the European Society of Hypertension (ESH) and of the European Society of Cardiology (ESC). J Hypertens. 2018;39(33):3021–3104.

2. Mistry EA, Mehta T, Mistry A, Arora N, Starosciak AK, De Los Rios La Rosa F, et al. Blood Pressure Variability and Neurologic Outcome after Endovascular Thrombectomy: A Secondary Analysis of the BEST Study. Stroke. 2020;511–8.

3. Geary RC. The Ratio of the Mean Deviation to the Standard Deviation as a Test of Normality. Biometrika. 1935;27(3/4):310.

4. Tobaldini E, Proserpio P, Oppo V, Figorilli M, Fiorelli EM, Manconi M, et al. Cardiac autonomic dynamics during sleep are lost in patients with TIA and stroke. J Sleep Res. 2019;(April):1–9.

5. Vollmer M. HRVTool - an Open-Source Matlab Toolbox for Analyzing Heart Rate Variability. 2019 Computing in Cardiology Conference (CinC). 2019;45:3–6.

6. Leite FS, Da Rocha AF, Carvalho JLA. Matlab software for detrended fluctuation analysis of heart rate variability. BIOSIGNALS 2010 - Proceedings of the 3rd International Conference on Bio-inpsired Systems and Signal Processing, Proceedings. 2010;225–9.

7. De Souza ACA, Cisternas JR, De Abreu LC, Roque AL, Monteiro CBM, Adami F, et al. Fractal correlation property of heart rate variability in response to the postural change maneuver in healthy women. Int Arch Med. 2014;7(1):1–7.

8. Tanaka H, Heiss G, McCabe EL, Meyer ML, Shah AM, Mangion JR, et al. Hemodynamic Correlates of Blood Pressure in Older Adults: The Atherosclerosis Risk in Communities (ARIC) Study. J Clin Hypertens. 2016;18(12):1222–7.

9. Hall JE, Granger JP, do Carmo JM, da Silva AA, Dubinion J, George E, et al. Hypertension: Physiology and pathophysiology. Compr Physiol. 2012;2(4):2393–442.

10. Bundy JD, Li C, Stuchlik P, Bu X, Kelly TN, Mills KT, et al. Systolic blood pressure reduction and risk of cardiovascular disease and mortality a systematic review and network meta-analysis. JAMA Cardiol. 2017;2(7):775–81.

11. Ebinger JE, Driver M, Ouyang D, Botting P, Ji H, Rashid MA, et al. Variability independent of mean blood pressure as a real-world measure of cardiovascular risk. EClinicalMedicine [Internet]. 2022;48:101442. Available from: https://doi.org/10.1016/j.eclinm.2022.101442

12. Tian J, Yuan Y, Shen M, Zhang X, He M, Guo H, et al. Association of resting heart rate and its change with incident cardiovascular events in the middle-aged and older Chinese. Sci Rep [Internet]. 2019;9(1):1–10. Available from: http://dx.doi.org/10.1038/s41598-019-43045-5

13. Palatini P, Reboldi G, Beilin LJ, Eguchi K, Imai Y, Kario K, et al. Predictive value of night-time heart rate for cardiovascular events in hypertension. The ABP-international study. Int J Cardiol [Internet]. 2013;168(2):1490–5. Available from: http://dx.doi.org/10.1016/j.ijcard.2012.12.103

14. Shaffer F, Ginsberg JP. An Overview of Heart Rate Variability Metrics and Norms. Front Public Health. 2017;5(September):1–17.

15. Farah BQ, Barros MVG, Balagopal B, Ritti-Dias RM. Heart rate variability and cardiovascular risk factors in adolescent boys. Journal of Pediatrics [Internet]. 2014;165(5):945–50. Available from: http://dx.doi.org/10.1016/j.jpeds.2014.06.065

16. Fang SC, Wu YL, Tsai PS. Heart Rate Variability and Risk of All-Cause Death and Cardiovascular Events in Patients With Cardiovascular Disease: A Meta-Analysis of Cohort Studies. Biol Res Nurs. 2020;22(1):45–56.

17. Schuster AK, Fischer JE, Thayer JF, Mauss D, Jarczok MN. Decreased heart rate variability correlates to increased cardiovascular risk. Int J Cardiol [Internet]. 2016;203:728–30. Available from: http://dx.doi.org/10.1016/j.ijcard.2015.11.027

18. Berger M, Solelhac G, Marques-Vidal P, Haba-Rubio J, Vollenweider P, Waeber G, et al. Association between nocturnal heart rate variability and incident cardiovascular disease events: The HypnoLaus population-based study. Heart Rhythm. 2022;19(4):632–9.

19. Usui H, Nishida Y. The very low-frequency band of heart rate variability represents the slow recovery component after a mental stress task. PLoS One. 2017;12(8):1–9.

20. Hayano J, Yuda E. Assessment of autonomic function by long-term heart rate variability: beyond the classical framework of LF and HF measurements. J Physiol Anthropol [Internet]. 2021;40(1):1–15. Available from: https://doi.org/10.1186/s40101-021-00272-y

21. Ernst G. Heart-rate variability—more than heart beats. Front Public Health. 2017;5(September):1–12.

22. Billman GE. The LF/HF ratio does not accurately measure cardiac sympatho-vagal balance. Front Physiol. 2013;4 FEB(February):1–5.

23. Hardstone R, Poil SS, Schiavone G, Jansen R, Nikulin V V., Mansvelder HD, et al. Detrended fluctuation analysis: A scale-free view on neuronal oscillations. Front Physiol. 2012;3 NOV(November):1–13.

24. Moon J, Choi KH, Park JH, Song TJ, Choi YS, Kim JH, et al. Sympathetic overactivity based on heart-rate variability in patients with obstructive sleep apnea and cerebral small-vessel disease. Journal of Clinical Neurology (Korea). 2018;14(3):310–9.

25. Albarado-Ibañez A, Arroyo-Carmona RE, Sánchez-Hernández R, Ramos-Ortiz G, Frank A, García-Gudiño D, et al. The role of the autonomic nervous system on cardiac rhythm during the evolution of diabetes mellitus using heart rate variability as a biomarker. J Diabetes Res. 2019;2019.

26. Rosenberry R, Nelson MD. Reactive hyperemia: A review of methods, mechanisms, and considerations. Am J Physiol Regul Integr Comp Physiol. 2020;318(3):R605–18.

27. McCrea CE, Skulas-Ray AC, Chow M, West SG. Test-retest reliability of pulse amplitude tonometry measures of vascular endothelial function: Implications for clinical trial design. Vascular Medicine. 2012;17(1):29–36.

28. Fantin F, Mattocks A, Bulpitt CJ, Banya W, Rajkumar C. Is augmentation index a good measure of vascular stiffness in the elderly? Age Ageing. 2007;36(1):43–8.

29. Nürnberger J, Keflioglu-Scheiber A, Opazo Saez AM, Wenzel RR, Philipp T, Schäfers RF. Augmentation index is associated with cardiovascular risk. J Hypertens. 2002;20(12):2407–14.
